# Transposable Element‐Mediated Structural Variation Drives Flower Colour Diversification in *Camellia*

**DOI:** 10.1111/pbi.70442

**Published:** 2025-11-06

**Authors:** Menglong Fan, Hong Jiang, Yuxiao Qu, Ying Zhang, Xinlei Li, Yan Wang

**Affiliations:** ^1^ Research Institute of Subtropical Forestry, Chinese Academy of Forestry Hangzhou, Zhejiang China; ^2^ Research Institute of Forestry, Chinese Academy of Forestry Beijing China

**Keywords:** *camellia*, flower colour, genome, structure variant, transposable element

## Abstract

The role of transposable elements (TEs) in genome evolution and phenotypic diversification in *Camellia* remains poorly understood. Here, we present an integrated analysis of genome resequencing data from 237 *Camellia* accessions and 11 de novo genome assemblies representing all major floral colour types. We constructed a comprehensive phylogenetic framework for the genus and suggest that the most recent common ancestor likely had white flowers. Comparative genomic analyses reveal structural variants across species that overlap with numerous transposable elements and contribute to genome content divergence. Using a graph‐based genome to characterise these structural variants, we find that lineage‐specific TE amplifications drive the regulatory network rewiring, which modulates homoeologous gene expression, influencing flower colour diversification. Further experimental validation identifies a lineage‐specific, high‐frequency presence variation mediated by a TIR transposon that regulates *MYB60* expression, suppressing anthocyanin biosynthesis and leading to large‐scale floral colour divergence. Therefore, these findings highlight the central role of TE‐mediated regulatory innovation in the evolution of flower colour in *Camellia* and offer broader insights into the molecular mechanisms driving phenotypic diversification in plants.

## 1 Introduction

Extensive genome‐wide association studies have revealed substantial genomic diversity underlying intraspecific phenotypic variation, including differences in coding sequences, gene expression and regulatory elements (Cheng et al. 2025; Fan et al. 2024; Gong et al. 2022). Beyond intraspecific analyses, studying phenotypic evolution across species under long‐term natural selection can uncover valuable genetic variations from wild gene pools (Wang et al. 2022), offering promising solutions to modern breeding bottlenecks. Such evolution often stems from sequence and expression changes driven by single nucleotide polymorphisms (SNPs) or structural variations (SVs). As a major source of genetic diversity, SVs—including insertions, deletions and inversions—play a pivotal role in plant evolution (Shi et al. 2024). Recent studies show they regulate key traits like flowering time, fruit quality and inflorescence meristem maintenance; (Wang et al. 2021; Chen, Wang, Kong, et al. 2023; Li et al. 2024).

Transposable elements (TEs) are abundant in plant and animal genomes. In some species, such as Camellia, maize and lily, TEs comprise over 80% of the genome (Hu, Fan, et al. 2024; Chen, Wang, Tan, et al. 2023a; Liang et al. 2025). TE insertions and movements drive SVs, including rearrangements, duplications and inversions, making them a key force in genome evolution (Zhang et al. 2025). TEs influence gene expression through multiple mechanisms: Introducing transcription factor binding sites, triggering epigenetic modifications (e.g., DNA methylation) (Li et al. 2024) and participating in alternative splicing to generate new transcript isoforms, potentially altering coding sequences, thereby providing raw material for the emergence of novel traits during evolution (Tian et al. 2025). Additionally, TE insertions in untranslated regions can posttranscriptionally modulate gene expression, contributing to phenotypic variation (Drongitis et al. 2019). Understanding how TE‐mediated genomic variation regulates phenotypic evolution is thus crucial for unravelling genome plasticity and trait development in plants.

The *Camellia* genus is an ideal model for studying TE‐driven phenotypic diversification. Comprising ~200 species—including ornamental camellias, tea and oil camellias—this Theaceae family member holds significant economic value and is primarily distributed across subtropical Asia (Zan et al. 2023). Genomic analyses show TEs constitute > 80% of the *Camellia* genome, coinciding with its remarkable genome‐wide phenotypic diversity (Zhang et al. 2021). However, this variation has also caused taxonomic ambiguity. Traditional morphology‐based systems divide the genus into 12–18 sections, yet inconsistencies persist (Chang 1998; Ming 1999; Sealy 1958). While molecular phylogenetic studies have clarified some taxonomic controversies, limited sampling and genomic data continue to hinder progress, impeding effective utilisation of the genus's genetic resources.

Among phenotypic traits, flower colour is an excellent model for studying evolution due to its visible variation and well‐defined biochemical basis. In *Camellia*, flower colour varies strikingly: Section *Camellia* species typically have red petals, Section *Chrysantha* species are yellow and many others bear white flowers—differences largely attributed to variations in flavonoid composition (Fan et al. 2022; Fan et al. 2023; Jiang et al. 2024). Here, we use *Camellia* flower colour to investigate TE's role in phenotypic evolution. Using whole‐genome data, we constructed a comprehensive phylogenetic framework and analysed flower colour evolution alongside geographic distribution. We generated high‐quality genomes for two *Camellia* species with contrasting petal colours and integrated them with nine published genomes to build a graph‐based pangenome, identifying structural variation across 172 population samples. Our analyses revealed that TE‐associated SVs drive gene expression divergence and genome evolution, providing new insights into the molecular mechanisms linking genomic dynamics to plant phenotypic diversity.

## 2 Result

### 2.1 Phylogenetic and Petal Colours Diversity

**FIGURE 1:**
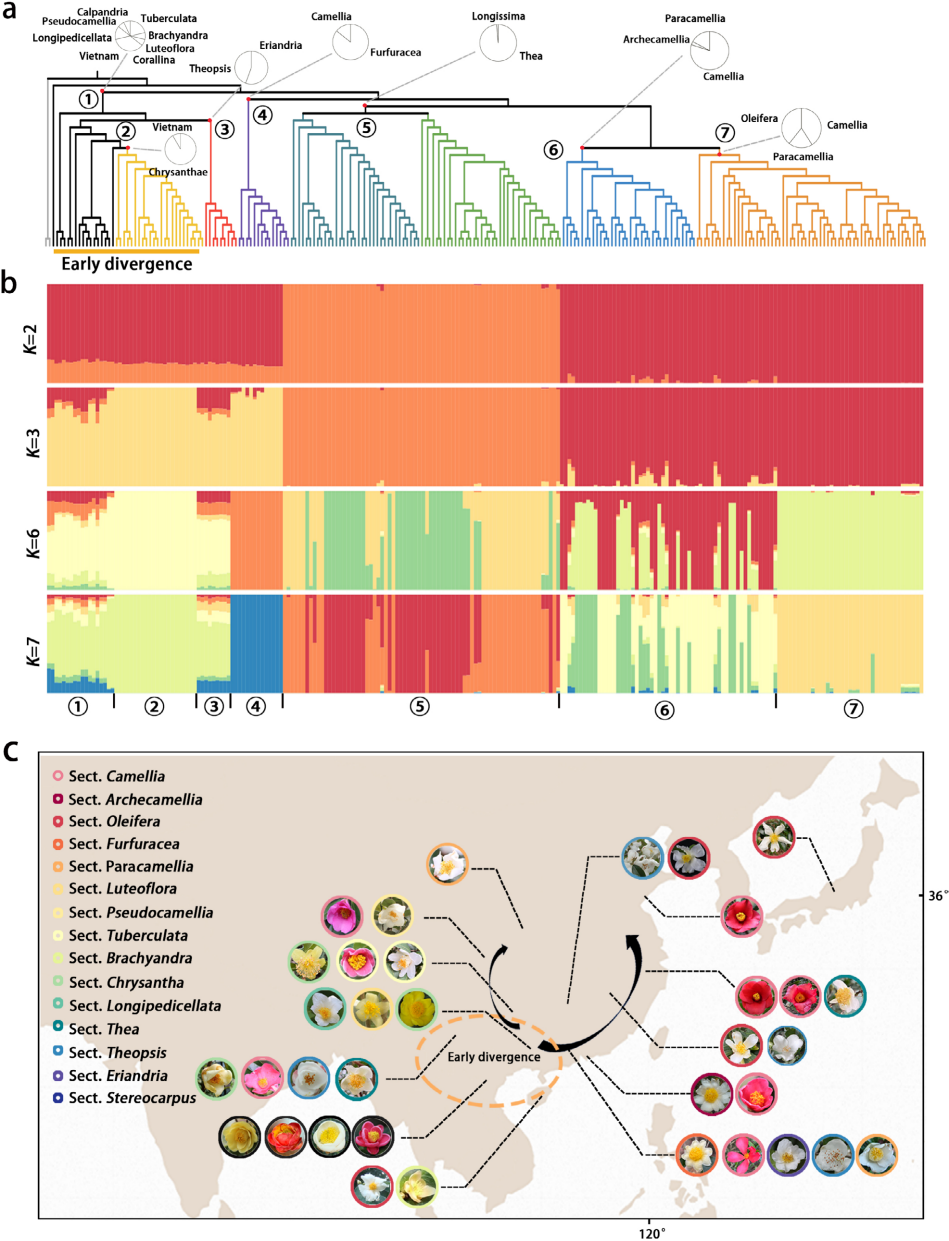
Phylogeny and phenotypic analysis of flower colour in *Camellia*. (a) Phylogenetic tree of 237 Camellia accession. Branch colours indicate seven major clades. Pie charts show the proportion of species from different sections within each clade. (b) Population structure of 237 Camellia accessions. The bar plots indicate membership proportions (*q*) for each accession at *K* = 2, 3, 6 and 7. (c) Geographic distribution and petal colour variation among 237 *Camellia* species. Representative flower colours are shown for species from each region. The yellow dashed area indicates the distribution of early‐diverging species.

To fully investigate the relationship between phylogeny and flower colour, we collected and sequenced diverse representative *Camellia* species genomes from China, Vietnam and Japan—the main regions of natural distribution. A total of 237 re‐sequencing datasets were used for further analysis, including 82 species newly sequenced in this study (Table S1). We first reconstructed the *Camellia* phylogenetic framework based on 4182517 single nucleotide polymorphism (SNP) (Figure 1a and Figures S1, S2), identifying seven well‐supported clades. Clade 1, comprising Sect. *Corallina*, *Calpandria*, *Brachyandra*, *Tuberculata*, *Longipedicellata*, *Pseudocamellia*, *Luteoflora* and most species from Vietnam are located at the base of the phylogenetic tree, indicating early divergence. These species are distributed in southwestern China and northern Vietnam, regions that may represent the origin of *Camellia*. Clade 2 is primarily composed of Sect. *Chrysantha* species and is closely related to Clade 1, also showing early divergence. Furthermore, concordant with the optimal solution of seven populations (K = 7), the phylogenetic tree topology was robustly corroborated by the results of population structure analysis and PCA analysis (Figure 1b and Figure S3). The extensive nucleotide sequence data enabled the reclassification of some taxa. *C. hongkongensis* appeared as an early‐derived species within Clade 4, which comprises Sect. *Furfuracea*, and shares traits such as a brown, rough capsule surface (similar to *C. furfuracea* ), red petals and oblong leaves.

Phenotypically, the phylogenetic tree assigns a unique basal position to *C. pilosperma*, bearing white petals. Clade 1, which diverged early, includes three flower colours—yellow, white and red—whereas later‐diverging clades lack the yellow type. Notably, yellow‐flowered *Camellia* species are restricted to the putative ancestral regions, while red‐ and white‐flowered species have a wider distribution (Figure 1c). The reconstruction of ancestral character states for flower colour was performed by using maximum parsimony and maximum likelihood methods; we obtained similar results, namely, that white colour was inferred as ancestral traits of *Camellia* (Figure S4). In addition, we also observed multiple potential transitions between red and white petals during evolution, such as within Clade 4, which includes red‐petaled *C. hongkongensis* and white‐petaled species from *Sect. Furfuracea*, as well as within the evolutionary branches of Sect. *Camellia* and Sect. *Paracamellia*.

### 2.2 Metabolic Basis of Red Floral Colour Variation

The petal coloration in *Camellia* species primarily includes varying shades of red, white and yellow. Our previous studies revealed that anthocyanins and flavanols are the main pigments in red and yellow‐like petals respectively (Fan et al. 2023; Fan et al. 2022; Jiang et al. 2024). To understand the metabolic basis underlying flower colour variation, UPLC‐MS analysis was conducted on 29 red‐like petal species from Sect. *Camellia*, Sect. *Paracamellia*, Sect. *Tuberculata* and Sect. *Longipedicellata*, covering all sections with red‐petaled species (Table S2). We identified 23 anthocyanins in red petals, with significant variation in their accumulation among species. Each species contained 2–15 anthocyanins. Cyanidin was detected in all analysed species and accounted for over 90% of the total anthocyanin content. Additionally, trace delphinidin amounts were also detected in *C. hongkongensis* , *C. rubituberculata* and *C. calcicola* . Notably, Cy3G*Ep*c was more abundant in purple‐red petals, while *Cy3G* predominated in red petals (Figure S5). These findings further support that flavonoid metabolite level differences form the biochemical basis for flower colour variation in *Camellia*.

### 2.3 High‐Quality Genome Assemblies of *Camellia* Species

To explore the relationship between genome evolution and flower colour diversity in *Camellia*, we analysed 11 diploid genomes from Sect. *Camellia*, *Paracamellia*, *Thea*, *Furfuracea* and *Chrysantha*, representing the three major flower colours (red, yellow and white). This included two endangered species ( *C. hongkongensis* and *C. chrysanthoides*) whose genomes were *de novo* assembled in this study, while the remaining genomes were obtained from previous studies (Chen, Wang, Kong, et al. 2023; Hu, Fan, et al. 2024; Shen et al. 2022; Wang et al. 2025). The two new genomes were sequenced using a combination of HiFi reads (average sequencing depth of 40× per genome), Illumina short reads (coverage depth of 70× per genome) and high‐throughput chromosome conformation capture data (Hi‐C, average coverage depth of 111× per genome). The final assembled genome sizes were 2.85 and 2.66 Gb, with contig N50 values of 88.44 megabase (Mb) and 86.78 Mb respectively—consistent with flow cytometry (Figure S6c,d) and survey analyses results (Figure S7a,b). A total of 99.5% and 99.9% of contigs were anchored to 15 chromosomes, with only 24 and 19 gaps remaining respectively (Table 1).

**TABLE 1:** Summary statistics from assembly and annotation of GH1 and JH3 genomes.

Species	*C. hongkongensis*	*C. chrysanthoides*
Assembly size (Gbp)	2.85	2.66
Anchor ratio (%)	99.5	99.9
Number of contigs	51	38
Contig N50 (Mbp)	88.44	86.78
Scaffold N50 (Mbp)	202.95	184.84
Number of gaps	24	19
Repeat ratio (%)	81.77	80.36
GC content (%)	38.88	38.66
Assembly completeness (BUSCO, %)	98.1	98.3
Merqury	50.8	53.31
Gene number	54 091	52 815
Annotation completeness (BUSCO, %)	96	96.9

Multiple genome assessments confirmed the high quality of the GH1 and JH3 assemblies. First, Benchmarking Universal Single Copy Orthologue (BUSCO) analysis revealed completeness scores of 98.1% and 98.3% respectively (Table 1). The consensus quality values (QVs) were 50.8 and 53.31 (Figure S7c,d), while clipping reveals assembly quality (CRAQ) analysis, which evaluates the regional and structural error rates, yielded regional assembly quality indicator (R‐AQI) scores of 99.42 and 98.16 and overall structural assembly quality indicator (S‐AQI) scores of 97.96 and 99.04, further supporting assembly accuracy. Additionally, Illumina read mapping achieved genome coverages of 99.92% and 99.96%. Hi‐C heatmaps also displayed strong diagonal signals, confirming proper spatial proximity of genomic regions (Figure S7e,f).

To minimise pipeline‐specific artefacts, we uniformly annotated all 11 genomes using a combined approach (ab initio, homology‐based and transcriptome‐based prediction). This identified 44 356–54 214 protein‐coding genes per genome, with BUSCO completeness ranging from 91.2% to 97.2% (Table S3). Over 93% of predicted genes had homologues in at least one major database (GO, KEGG, COG, Pfam, Swiss‐Prot, TrEMBL, InterProScan or NR).

### 2.4 TE Expansion Effect Genome Architecture in *Camellia*

**FIGURE 2:**
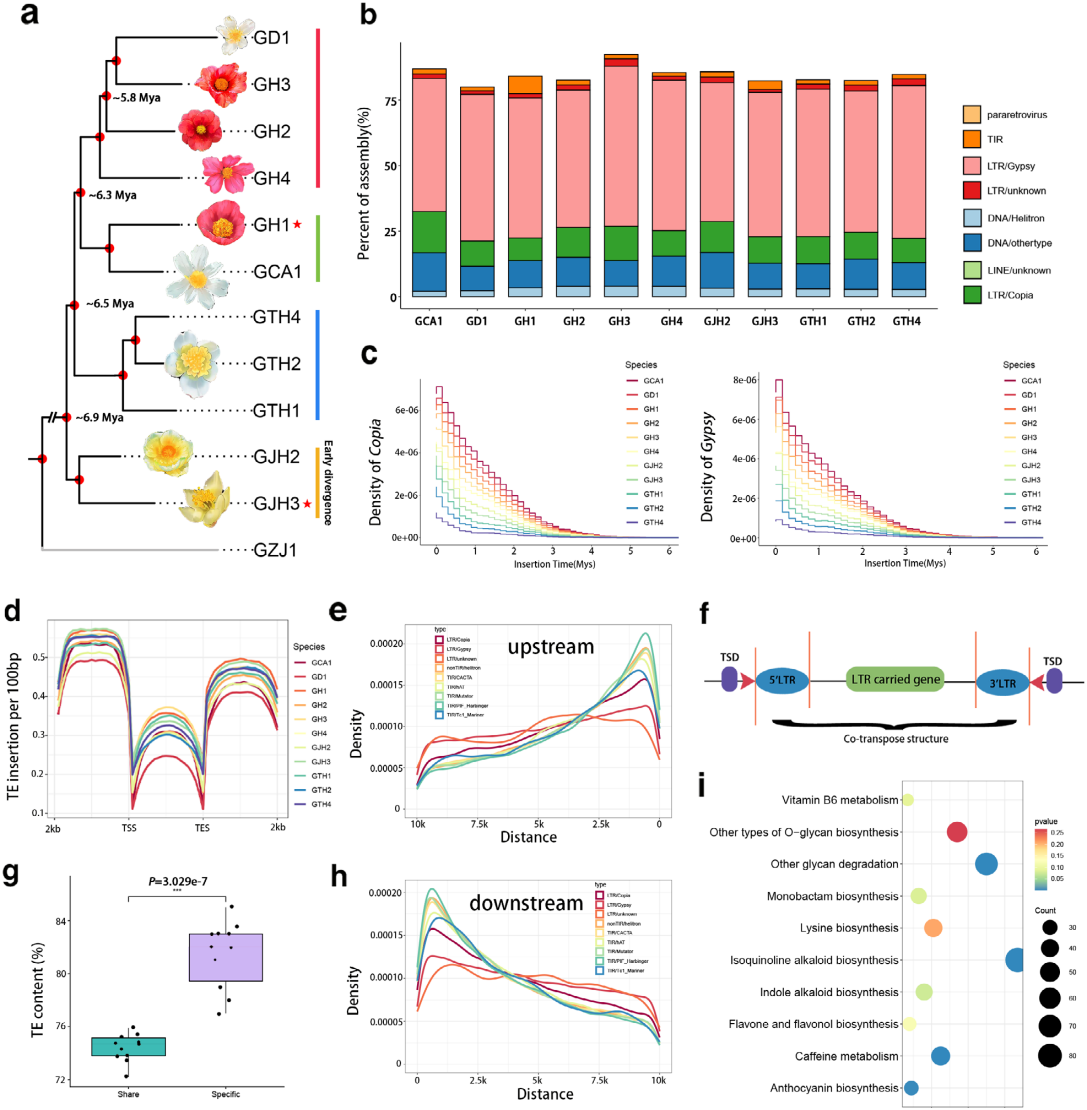
Transposable element (TE) features in *Camellia* genomes. (a) Phylogenetic tree inferred from single‐copy orthologs using ASTRAL. (b) Bar plot showing the proportion of different TE types. (c) Insertion time distribution of full‐length *Copia* and *Gypsy* retrotransposons. (d) TE density per 100 bp in gene bodies and 2 kb flanking regions across 11 Camellia genomes. (e, h) Distribution of different TE types in 10 kb flanking regions of genes. (g) The comparison of TE proportion in shared and species‐specific genomic regions. (f, i) Schematic of a gene‐containing LTR retrotransposon and the top 10 significantly enriched KEGG terms of these genes.

A species tree constructed using single‐copy orthologous genes revealed phylogenetic relationships among the 11 genomes consistent with those observed in the population data (Figure 2a). TEs accounted for 76.6%–84.5% of the sequences in these genomes (Table S4). Long terminal repeat retrotransposons (LTRs), particularly Gypsy elements, were the most abundant, each comprising over 47% (Figure 2b). To investigate the role of TEs in the genomic evolution of the *Camellia* genus, we analysed their insertion times and distributions across the phylogeny. Between 10 194 and 24 196 full‐length LTRs (fl‐LTRs) were identified, with evidence of continuous *Copia* and *Gypsy* expansion over the past 6 million years (Figure 2c). TEs were mainly distributed within 2 kb surrounding gene bodies (Figure 2d), though *Gypsy* elements showed a broader range, primarily between 2 and 5 kb (Figure 2e,h), suggesting a wider involvement in gene regulation. TE content was significantly higher in species‐specific regions than in shared (homologous) regions (Figure 2g), highlighting their role in genome differentiation. Moreover, closely related species shared a highly similar number of fl‐LTRs (Figure S8b), consistent with the general understanding in evolutionary biology. On average, each genome contained approximately 11 400 TE‐associated genes (Figure 2f), with significant enrichment (*p* < 0.05) in some Kyoto Encyclopedia of Genes and Genomes (KEGG) terms related to secondary metabolism, including ‘Caffeine metabolism,’ ‘Isoquinoline alkaloid biosynthesis’, ‘Anthocyanin biosynthesis’ and ‘Flavone and flavonol biosynthesis’ (Figure 2i). These findings underscore the crucial role of TEs in driving metabolic diversity and stress response evolution.

**FIGURE 3:**
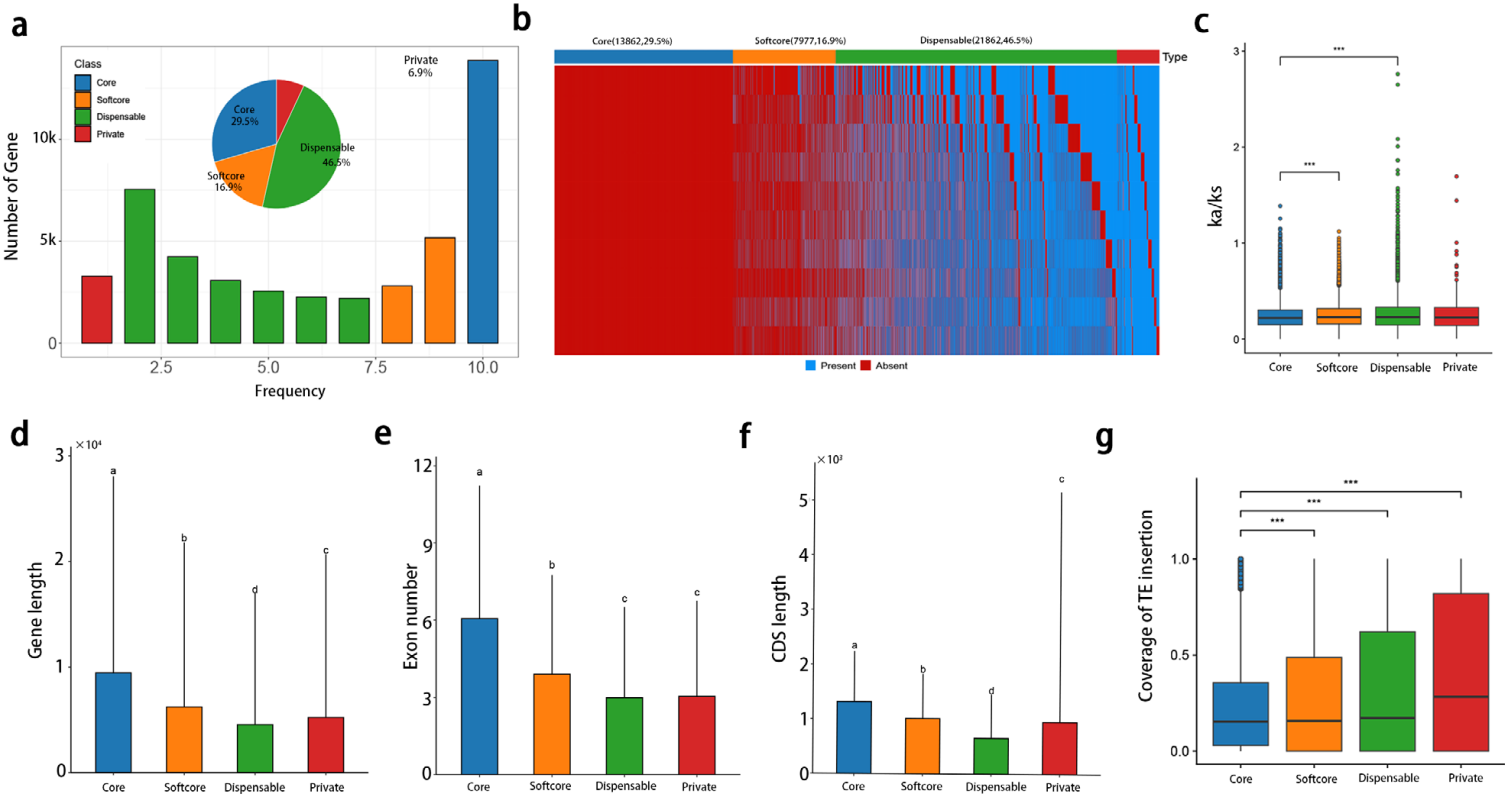
Pan‐gene analysis across Camellia genomes. (a) The histogram showing the frequency distribution of pan‐gene families across genomes. The pie chart shows the proportion of different pan‐gene family types. (b) Presence/absence variation of all pan‐gene families in 11 genomes. (c–g) The comparisons of *Ka/Ks* values, gene length, exon number, CDS length and TE insertion numbers in genes. Different letters indicate significant differences at *p* < 0.05. *** represent significant differences (*p* < 0.001).

To further investigate the impact of TEs on gene evolution after species formation, we classified genes from 11 species/varieties into four categories using previously reported strategies (Fang et al. 2024; Yang et al. 2022). A total of 46 979 gene families were grouped as Core (13 862, 29.5%), Softcore (7977, 16.9%), Dispensable (21 862, 46.5%) and Private (3278, 6.9%). The proportion of each gene category was similar across species, with average counts of 22 260 (43.0%), 12 448 (24.0%), 13 299 (25.4%) and 3933 (7.4%) respectively (Figure 3a,b). Compared with core genes, dispensable and private genes showed higher nonsynonymous‐to‐synonymous substitution ratios (*Ka/Ks*) (Figure 3c), shorter gene and coding sequence lengths, fewer exons (Figure 3d–f) and more TE insertions (Figure 3g), suggesting that the accumulation of TE is a characteristic of the dispensable genome compartment. Therefore, these results further support the role of TEs in genome divergence within the *Camellia* genus.

## 3 TEs Contribute to Most Structural Variations

**FIGURE 4:**
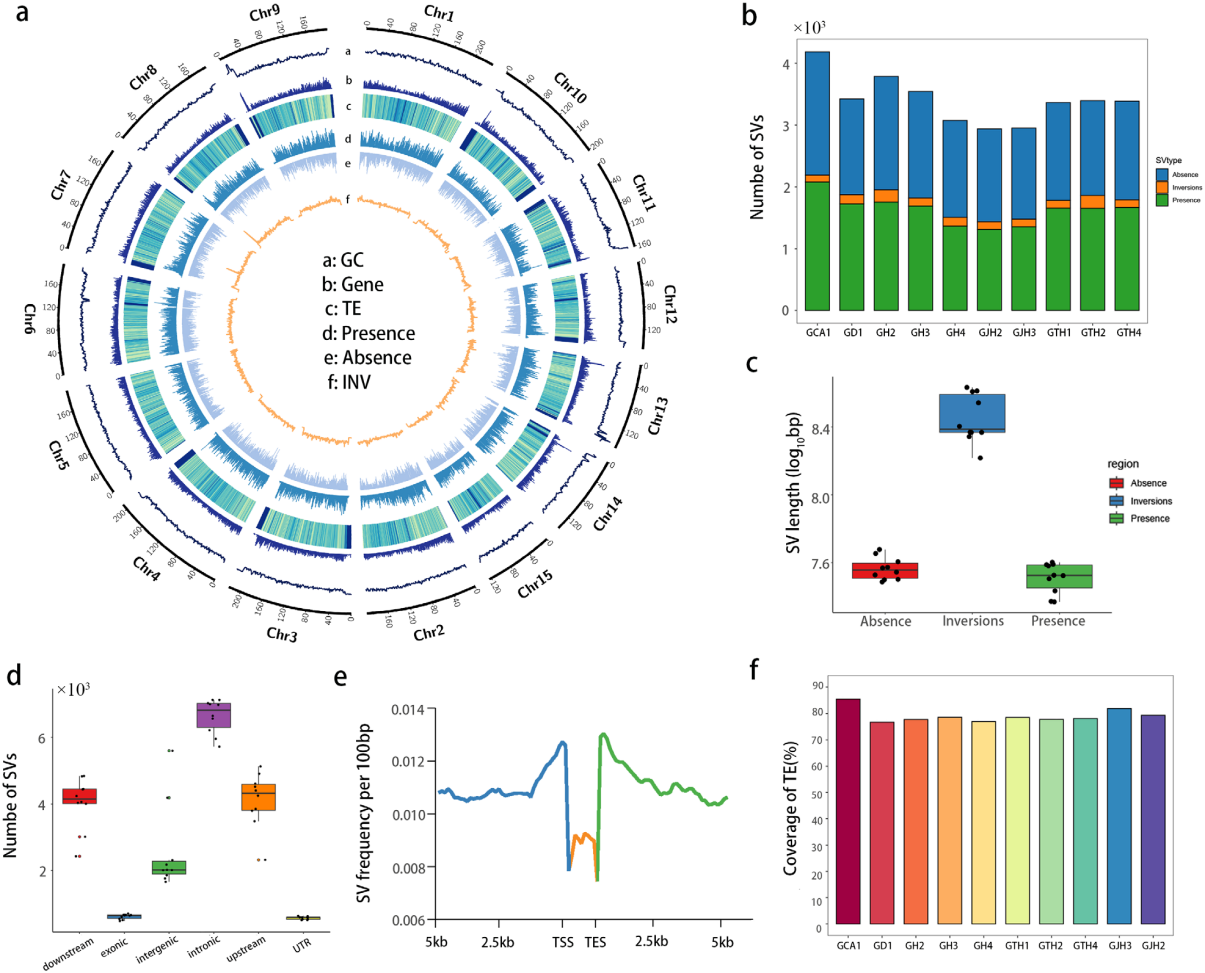
Characteristics of structural variations among 11 Camellia genomes. (a) The distribution of GC content, gene count, TE density and SV number in the GH1 reference genome. (b) The number of different SV types across Camellia genomes. (c) Total SV size in each genome, including presence/absence variations and inversions. (d) SV counts in six genomic regions: Downstream (+2 kb), exon, intergenic, intron, upstream (−2 kb) and UTR. (e) SV density per 100 bp in gene bodies and 5 kb flanking regions. (f) Proportion of SVs overlapping with TEs.

To explore large‐scale genomic variations among *Camellia* species, we aligned the 11 chromosome‐level genomes to the GH1 reference. Consistent with the phylogenetic tree, *C. crapnelliana* and *C. hongkongensis* exhibited a higher number of syntenic regions, indicating a closer relationship (Figure S9). In total, 326 392 SVs were identified, including presence/absence variations (PAVs) (24 bp to 1.501 Mb) and 15 074 inversions (100 bp to 71.462 Mb), accounting for 12.2% to 25% of each genome (Figure 4a–c). The numbers of presence and absence variations were nearly equal (Table S5). Most SVs were located in intronic and upstream/downstream regions (> 23%), while significantly fewer were found in exonic and UTR regions (Figure 4d), suggesting that SVs affecting coding regions may be under stronger selective pressure. Notably, SVs were mainly concentrated within 2 kb upstream and downstream of genes (Figure 4e), and on average, 79.1% overlapped with TEs, with *Gypsy* retrotransposons being the most abundant type, accounting for an average of 45% of all TE‐associated SVs (Figure 4f and Figure S10), highlighting the central role of TEs in driving structural variation. We randomly selected 25 SVs for manual validation. These SVs were confirmed by long reads validation (Figure S11).

### 3.1 Structural Variations Affect Orthotonus Expression in Flower

**FIGURE 5:**
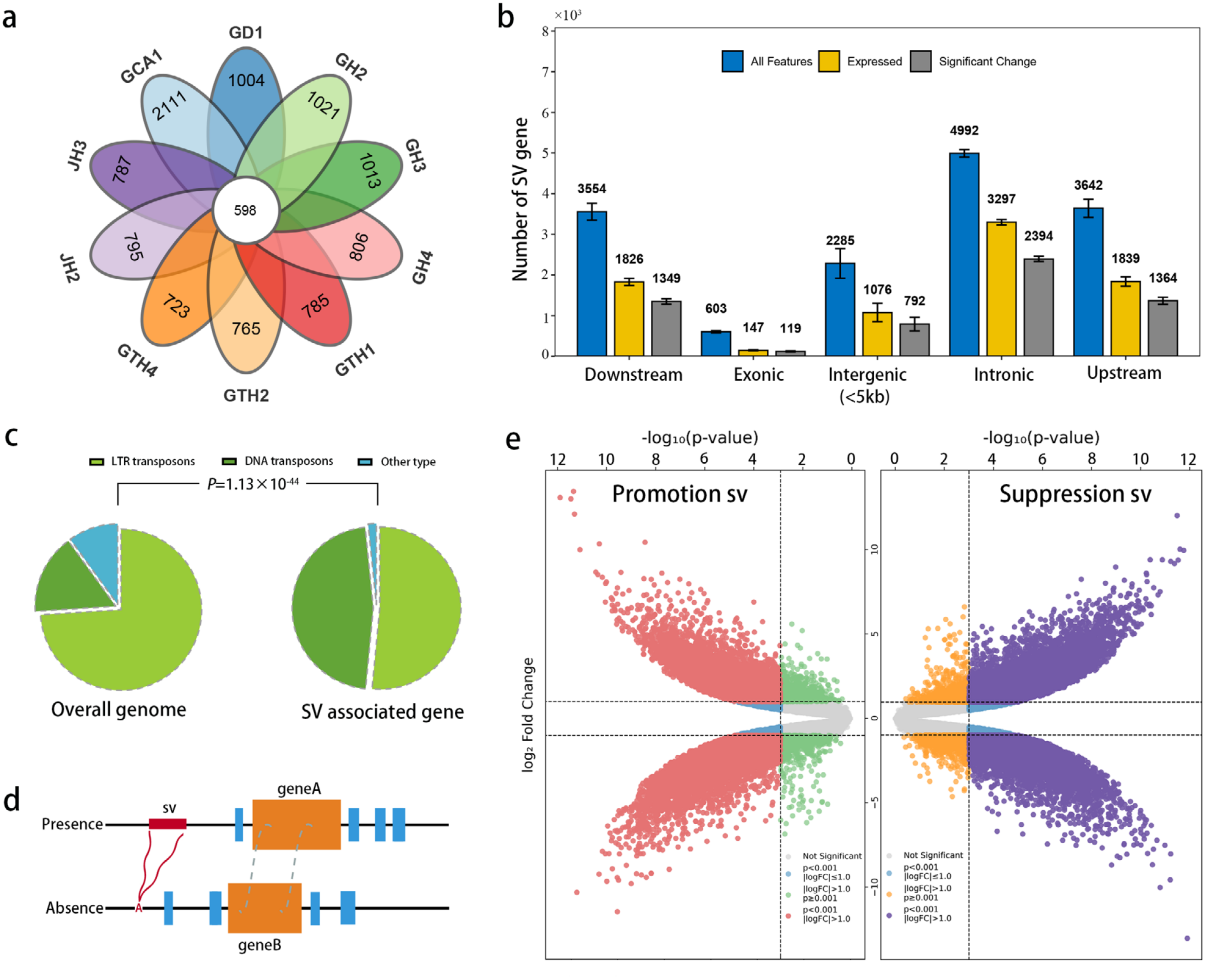
The effect of SVs on gene expression. (a) Venn diagram showing overlap of SV‐associated genes across 10 *Camellia* genomes. Distinct petals represent unique SV genes; intersecting regions represent shared SV genes. (b) Expression of SV genes by SV location type. (c) Proportion of different TE types in expression‐associated SVs. (d) Schematic representation of presence/absence SV genotype groups. (e) The expression fold‐change of SV genes between the presence and absence genotype groups.

We identified 37 175 SV genes, defined as the nearest genes located within 10 kb of an SV. Among these, only 598 SV genes were shared across species—markedly fewer than species‐specific SV genes (Figure 5a). Notably, more SV genes were shared among closely related species, suggesting that SVs may drive species divergence by altering gene structure or expression. To explore the effects of SVs on the transcription of orthologous genes in floral tissues, we analysed petal transcriptomes from 10 *Camellia* species/variants; these SV genes included 25 652 genes expressed in petals, and 24 950–26 388 collinear genes were used for subsequent analyses. Based on SV insertion sites, these genes were classified as exonic, intronic, upstream, downstream or intergenic. Differential expression analysis revealed that a substantial proportion of SVs influenced gene expression. Specifically, an average of 81% of SVs in coding regions affected expression, compared to 73% for intergenic SVs (Figure 5b). Among the SVs impacting gene expression, 98.8% were TE‐derived, with DNA transposons accounting for 40.3%, despite comprising only 12.4% of the genome's TE content (Figure 5c). A chi‐square test confirmed that this enrichment was highly significant (*p* = 1.1357 × 10^−44^), underscoring the prominent role of DNA transposons in gene regulation.

Following established methods, longer sequences (genomes with SV insertions) were classified as present, and shorter sequences (genomes with SV deletions) as absent. SV genes were grouped accordingly into presence and absence categories (Figure 5d). Expression levels of collinear genes in these groups were compared, with fold changes in affected SV genes ranging from −12.42 to −13.45 (Figure 5e). Additionally, a binomial test showed no significant bias toward either upregulation or downregulation of gene expression in petals (*p* = 0.06). In summary, these results highlight the complex and significant role of SVs in regulating petal gene expression.

### 3.2 SVs Associated With Flower Colour Diversification

**FIGURE 6:**
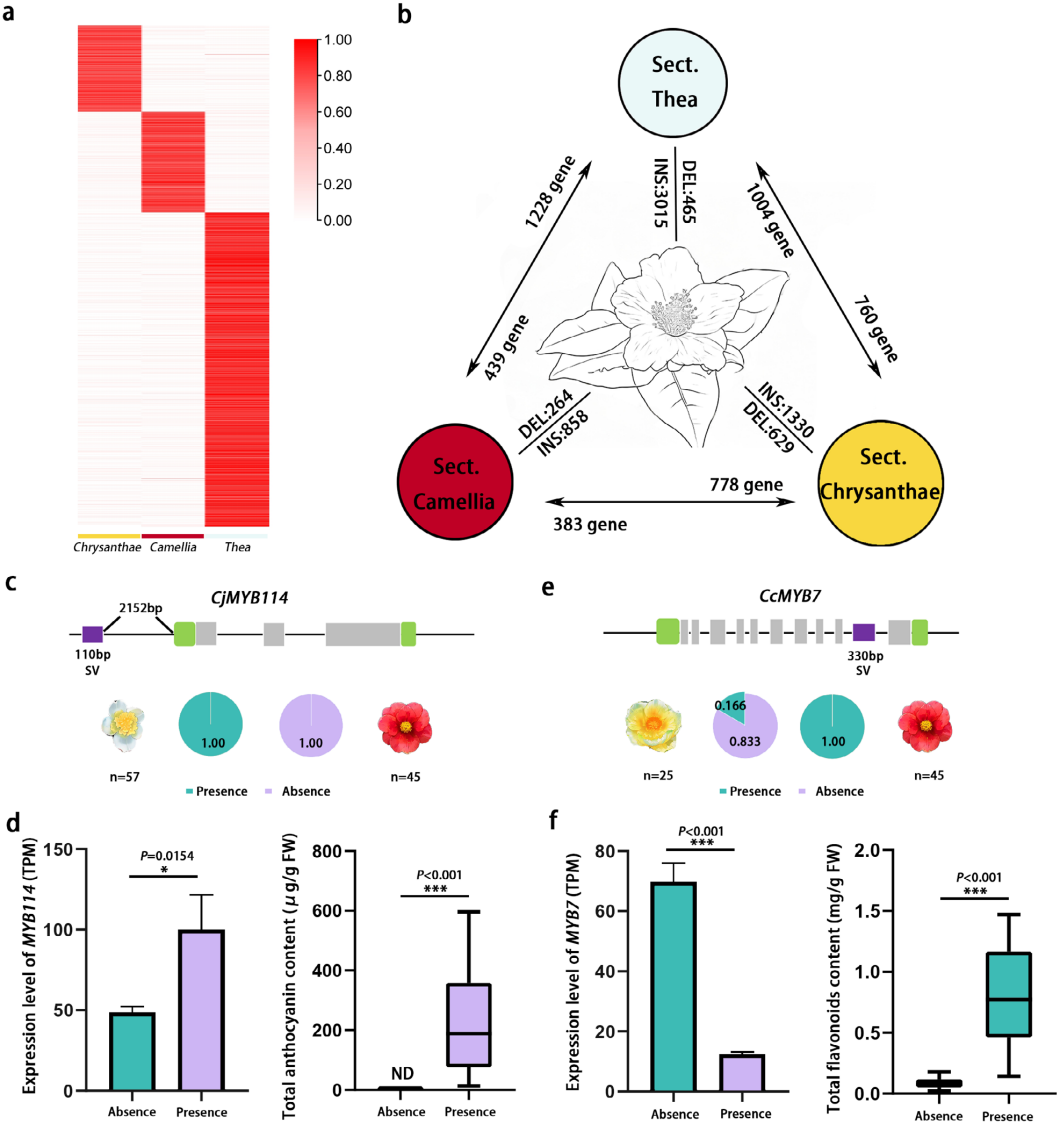
Structural variations potentially linked to flower colour divergence. (a) Heatmap showing high‐frequency SVs in Sect. *Thea*, Sect. *Camellia* and Sect. *Chrysantha*. Red and white indicate high and low frequency respectively. (b) The difference of SV and SV‐associated gene expression across three subgroups. (c) The frequency of accessions with presence or absence SV (associated with *MYB114*) genotype for Sect. *Camellia* and Sect. *Thea* accessions. (d) The comparison of *MYB114* expression level and anthocyanin content between SV presence and absence species. (e) The frequency of accessions with the presence or absence SV (associated with *MYB7*) genotype for Sect. *Camellia* and Sect. *Chrysantha* accessions. (f) The comparison of *MYB7* expression level and flavonoid content between SV presence and absence species.

To investigate the role of SVs in the flower colour diversification in *Camellia* species, we merged 326 392 presence/absence variations (PAVs) identified across all species into a nonredundant set of 207 853 SVs for analysis. A total of 176 individuals (45 red‐flowered, 25 yellow‐flowered and 106 white‐flowered samples from Sect. *Thea*, Sect. *Camellia*, Sect. *Chrysantha* and Sect. *Paracamellia*) were mapped to the graph genome, yielding 194 471 SVs across the population. The phylogenetic tree constructed from SV sets showed a topology largely consistent with SNP‐based results (Figure S12). We identified 3337 (Sect. *Chrysantha*), 3659 (Sect. *Camellia*) and 11 419 (Sect. *Thea*) subgroup‐specific high‐frequency SVs (frequency > 0.8 in the target subgroup and < 0.2 in others) in the three major subgroups (Figure 6a), involving 6152 SV genes (Figure 6b). The floral colour diversity observed among these species can be attributed to variations in both the composition and abundance of flavonoid compounds. To investigate the genetic basis of this variation, we identified key structural genes involved in the flavonoid biosynthetic pathway across these genomes by screening for conserved functional domains. No obvious gene losses were detected among the structural genes. Interestingly, no structural variants (SVs) were detected within the exonic regions of these key structural genes; however, SVs were identified within promoter regions (up to 2 kb upstream) of several core structure genes, including *DFR*, *ANS* and *FLS*. These findings suggest that SVs may influence flavonoid biosynthesis by directly or indirectly modulating the expression of structural genes.

Using petal transcriptomic data from 10 white‐, 5 yellow‐ and 8 red‐flowered species, we compared the expression of these genes and found 3503 with significant differential expression among colour types (FDR < 0.05, fold change > 1). Notably, 643 genes were upregulated in red petals; for example, *MYB114* has been reported as a transcriptional activator of anthocyanin biosynthesis (Zhang et al. 2024). In Camellia species from the *red‐flowered* section, *MYB114* exhibits higher expression levels in red petals, despite showing minimal differences in its coding sequence. However, a structural variant involving the insertion of a TIR‐type transposable element approximately 2.1 kb upstream of the gene was identified exclusively in these species. In contrast, white‐flowered species from Sect. *Thea*, which typically lack this structural variant, show markedly lower expression of *MYB114* in their petals (Figure 6c,d). In addition, we identified 778 structural variant (SV)‐associated genes that were differentially expressed between yellow and red petals. For instance, a high‐frequency SV specific to Sect. *Chrysantha* was detected within the ninth intron of a *MYB7* ortholog (Figure 6e). This SV consists of a 330 bp insertion comprising an LTR/Copia‐type transposable element. In *Arabidopsis thaliana* , *AtMYB7* functions as a negative regulator of flavonol biosynthesis. Compared to genotypes lacking this SV, the expression of the *MYB7* ortholog was significantly reduced in accessions carrying the insertion (Figure 6f). In addition, 388 were specifically upregulated in white petals, potentially suppressing anthocyanin biosynthesis. These findings provide important insights into the molecular mechanisms underlying flower colour evolution in *Camellia*.

**FIGURE 7:**
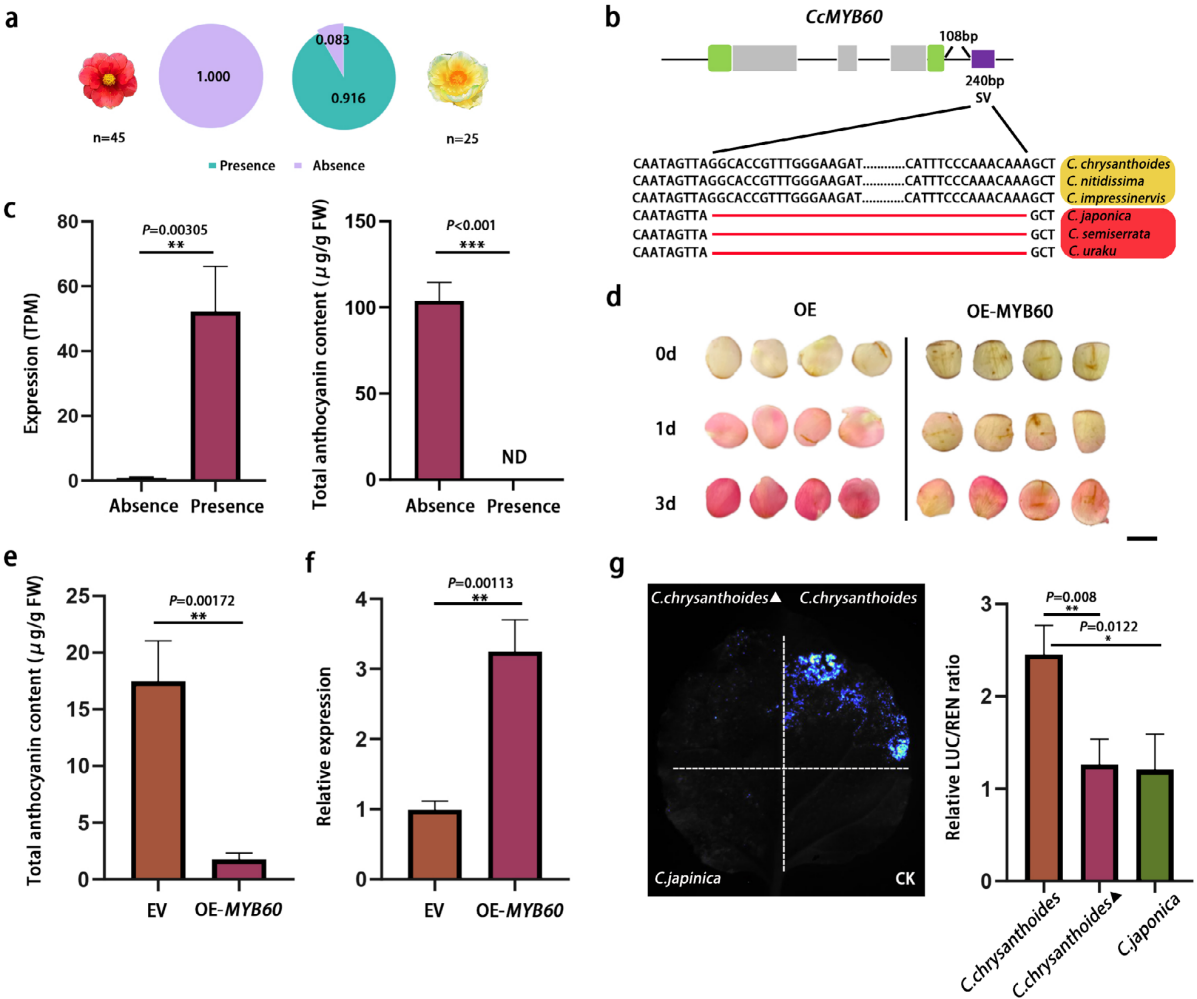
An SV potentially associated with flower colour. (a) The frequency of accessions with presence or absence SV (associated with *MYB60*) genotype for Sect. *Camellia* and Sect. *Chrysantha* accessions. (b) Schematic diagram of SV affecting the *MYB60* gene in yellow and red‐flowered *Camellia* species. (c) The comparison of *MYB60* expression level and anthocyanin content between SV presence and absence species. (d) Phenotypes of camellia petals overexpressing *CcMYB60*. Scale bar: 1 cm. (e) Anthocyanin content in *MYB60*‐overexpressing camellia petals. Data are mean ± SD (*n* = 3). Asterisks indicate significant differences (Student's *t*‐test). (f) Relative expression of *MYB60* in *MYB60*‐overexpressing camellia petals. Data are mean ± SD (*n* = 3). Asterisks indicate significant differences (Student's t‐test). (g) The activity of LUC constructs with or without the 240 bp SV region of *MYB60* in transfected *N. benthamiana*. Each bar represents the mean ± SD from three independent experiments.

Another high‐frequency presence SV was specific to Sect. *Chrysantha* (predominantly yellow‐flowered) but absent in Sect. *Camellia* (predominantly red‐flowered). This SV corresponds to a 240 bp insertion of a TIR‐type transposable element, located 108 bp downstream of an *MYB60* transcription factor ortholog (Figure 7a,b). *MYB60* expression was significantly higher in yellow‐flowered species than in red‐flowered ones (Figure 7c), and transient expression assays confirmed its strong suppression of anthocyanin biosynthesis in petals (Figure 7d–f). To test the effect of SV, we cloned gene and downstream regions of *MYB60* from *C. japonica* (TE‐absent), *C. chrysanthoides* (TE‐present) and *C. chrysanthoides* ^▲^ (TE‐absent), fusing them to a LUC reporter. Dual‐luciferase assays showed that the fragment with the SV had significantly higher LUC/REN activity than those without it, confirming the SV's positive regulatory role in *MYB60* expression (Figure 7g). These findings provide strong evidence for TE‐mediated complex regulation of homologous gene expression.

## 4 Discussion

Understanding how genome evolution shapes phenotypic diversity is a key goal in evolutionary biology. Increasing evidence indicates that TEs are more dynamic and functionally important than once believed (Tian et al. 2025; Wang et al. 2021). The genus *Camellia*, known for its high TE content and striking phenotypic variation, now has genome assemblies for over 10 species. Using these resources alongside population‐scale sequencing data, we identify conserved genomic features across lineages and phenotypes, including TE‐mediated sequence composition differences and expression divergence of orthologous genes.

Due to the genus's high species diversity and extensive morphological variation, species identification and classification within *Camellia* remain challenging. In this study, we analysed genome sequences from 237 accessions representing 129 species, covering all sections recognised in *Flora of China* (Chang 1998), 13 sections from Monograph of the Genus Camellia (Ming 1999) and 11 sections in the Sealy classification system (Sealy 1958), filling gaps in previous phylogenetic research. Using whole‐genome resequencing data, we conducted a comprehensive phylogenetic analysis. Our results show that *C. hongkongensis* and species from Sect. Furfuracea form a single clade, rather than clustering with Sect. *Camellia*, where they were previously placed by Chang and Ming. Notably, their semi‐persistent tepals and distinct three‐style morphology are atypical for Sect. *Camellia*. Additionally, we found that *C. rubriflora* is nested within Sect. *Chrysantha*, despite its solitary red flowers on young shoots—contrasting with the yellow flowers on mature branches typical of the section. However, it shares leathery, glabrous, oblong‐elliptic leaves with other Sect. *Chrysantha* species. Similarly, *C. longissima* falls within Sect. *Thea*, conflicting with traditional classifications by Chang (1998) and Ming (1999). Chang placed it and *C. hekouensis* in a separate Sect. *Longissima*, while Ming grouped them with *C. longipetiolata* in Sect. *Longipedicellata*. Recent phylogenomic studies also indicate that Sect. *Longissima* is not monophyletic and is nested within Sect. *Thea* (Zhang et al. 2023). These cases highlight the limitations of traditional taxonomy in the face of extensive morphological convergence and variability.

A well‐resolved phylogeny provides a robust foundation for studying phenotypic evolution. Based on our phylogenetic, ancestral state reconstruction and distribution patterns, we infer that the most recent common ancestor of *Camellia* likely had white petals. At the genus level, an evolutionary trend is clear: gradual loss of yellow pigmentation followed by the emergence of red and pink flowers. Intriguingly, a recent study suggests a similar transition has occurred within *Rosa*, which similarly began diversifying around 6 million years ago (Cheng et al. 2025). This parallel pattern implies convergent natural selection on floral colour preference in both genera. Flower colour is primarily determined by petal pigments (Hu, Chen, et al. 2024). Our analysis across *Camellia* species revealed that anthocyanins are the main chromogenic compounds for red and pink petals. Previous work showed that yellow pigmentation in Sect. *Chrysantha* stems from flavonol derivatives rather than carotenoids (Jiang et al. 2024), suggesting that flavonoid biosynthesis genes play a central role in *Camellia* floral coloration. Using a domain‐based search strategy, we identified key structural genes in flavonoid biosynthesis across these genomes (Du et al. 2024), with no loss of core enzymatic genes. Thus, regulatory changes—such as cis‐regulatory element rearrangements, transcriptional rewiring and epigenetic modifications—likely drive shifts in structural gene expression and may explain floral colour transitions in *Camellia*. Additionally, we cannot exclude potential contributions from single nucleotide polymorphisms (SNPs) and small insertions and deletions (Indels).

Our genomic analysis of *Camellia* revealed that structural variants (SVs) can either activate or repress gene expression, consistent with findings in other plants (Chen, Wang, Kong, et al. 2023; Li et al. 2024). TEs are a major source of SVs and play a key role in shaping regulatory networks across plant and animal genomes (Wang et al. 2021; Wang et al. 2022). Most SVs in our study overlapped with TEs, primarily LTR retrotransposons, while DNA transposons were significantly enriched among SVs linked to expression changes. A similar enrichment of DNA transposons upstream of species‐specific genes was reported in *Gossypium* (Tian et al. 2025), suggesting that DNA transposon polymorphisms contribute to genome divergence and homoeolog gene expression divergence. In tomato, TE polymorphisms have also driven phenotypic variation during domestication (Domínguez et al. 2020).

Using a graph‐based pangenome and population resequencing data, we identified lineage‐specific high‐frequency SVs across *Camellia* lineages. Integrating petal transcriptomics revealed candidate genes for floral colour variation, where expression divergence likely stems from SVs. MYB transcription factors regulate flavonoid metabolism, development and stress responses (Wang et al. 2020; An et al. 2020). Our SV gene set included key flavonoid regulators like *MYB114* and *MYB7* (He et al. 2023; Wang et al. 2017). Interestingly, the intronic insertion in *MYB7* potentially encodes miR396, which has been reported to regulate floral development by targeting growth‐regulating factors (GRFs) (Yuan et al. 2020), its regulatory mechanisms in *Camellia* require further investigation and will be the subject of upcoming work. In addition, *AtMYB60*—a negative anthocyanin biosynthesis regulator in lettuce; its orthologs had a *Chrysantha*‐specific TIR type transposon insertion downstream, and transient assays suggested *CcMYB60* suppress anthocyanin biosynthesis, possibly suppressing anthocyanins to yield yellow flowers. In Brassica, SVs can enhance expression by introducing new transcription factor binding sites. Similarly, we found HD‐ZIP and WRKY transcription factors enriched in SV regions, implying TEs remodel regulatory networks via novel binding motifs, driving *Camellia* floral colour divergence. Our functional validation supports TE‐mediated SV regulation of homoeolog expression as a key driver of intra‐generic phenotypic diversification.

## 5 Conclusion

In summary, by integrating high‐quality genome assemblies with extensive population‐scale genomic data, we elucidated SVs' role in *Camellia* flower colour evolution. Our study provides a comprehensive genus‐wide genetic and transcriptomic landscape, identifying lineage‐specific, high‐frequency SVs underlying phenotypic divergence. We show that TE‐mediated SVs likely introduce regulatory network rewiring, modulating homologous gene expression and fuelling transcriptional divergence during the evolutionary diversification of *Camellia* species.

## 6 Material and Method

### 6.1 Plant Materials Collection

Genome sequencing data were collected for 237 *Camellia* germplasm accessions, including 82 species sequenced for the first time. These resources were collected over two decades across nearly the entire natural distribution range of the genus, mainly in China, Vietnam and Japan. All accessions are conserved at the *Camellia* Germplasm Resource Center, Research Institute of Subtropical Forestry, Chinese Academy of Forestry. They represent all 18 sections of *Camellia* in the *Flora of China* (Chang 1998) and exhibit broad phenotypic diversity, including 47 samples from Sect. *Camellia*, 21 samples from Sect. *Chrysantha*, 72 from Sect. *Thea*, 10 from Sect. *Furfuracea*, 1 from Sect. *Archecamellia*, 8 species from Vietnam and others. Sample details are provided in Table S1.

### 6.2 Genome Sequencing

Genomic DNA was extracted from tender leaves using the cetyltrimethylammonium bromide (CTAB) method. For next‐generation sequencing, 150 bp paired‐end reads were generated on the DNBSEQ‐T7 platform (Berry Genomics), yielding ~180 Gb per sample, with QV30 scores exceeding 94%. For circular consensus sequencing (CCS), PacBio HiFi libraries were prepared per manufacturer protocols (Pacific Biosciences) and sequenced on the Revio or Sequel II platforms, generating ~108.5 Gb of high‐fidelity (HiFi) reads per sample after quality control. Hi‐C libraries were constructed using the Illumina TruSeq DNA Sample Prep Kit and sequenced on the Illumina HiSeq platform, producing ~300 Gb per sample. To enhance genome annotation, multiple tissues (roots, stems, young and mature leaves, flowers and fruits) were collected from GH1 and GJH3, pooled and subjected to CCS‐based transcriptome sequencing, with each composite sample yielding over 100 Gb of data.

### 6.3 Genome Assembly, and Evaluation

Genome size for the two newly sequenced species was estimated using the *k*‐mer frequency method with Jellyfish (v2.3.0; Marcais and Kingsford 2011) and findGSE (Sun et al. 2018). Furthermore, flow cytometry analysis was also performed to validate genome size. Contig‐level genome assembly was conducted using Hifiasm (v0.24.0‐r703) with parameters ‘−l 3 −z 20’ (Cheng et al. 2021). The redundant contigs were removed using the Khaper algorithm with default settings (Chen, Wang, Kong, et al. 2023). For pseudomolecule construction, Hi‐C paired‐end reads were aligned to the respective contigs using bwa‐mem2 (v2.2.1) with the −5SP parameter (Langarita et al. 2023). The resulting BAM files and primary contigs were processed through the Haphic (v1.0.6) pipeline to generate scaffold assemblies (Zeng et al. 2024). Misassemblies and chimeric errors were corrected by manual curation using Juicebox (v1.11.08) (Robinson et al. 2018).

Genome assembly quality was assessed using multiple approaches. The genome completeness was evaluated using 1614 single‐copy orthologs from the Embryophyta_odb10 database (Seppey et al. 2019). Quality values were estimated with Merqury (v.1.3) to determine base‐level accuracy (Rhie et al. 2020). Chimeric contigs and local misassemblies were detected using CRAQ (v.1.0.9) (Li et al. 2023). Additionally, short reads and HiFi reads were mapped to the genome to assess assembly consistency.

### 6.4 Transposable Element and Protein‐Coding Gene Annotation

For TE annotation, the EDTA pipeline—which integrates structural and homology‐based methods—was used with the ‘‐sensitive 1’ parameter (Ou et al. 2019). For ‘LTR/unknown’ type transposons, TEsorter was used for reclassification (Zhang et al. 2022). In addition, transposable elements sharing over 90% sequence identity are defined as shared transposable elements. The same pipeline was applied for gene structure annotation in each genome, combining transcript‐based, homology‐based and *de novo* prediction methods. Briefly, PacBio mRNA sequencing was performed on mixed samples of root, stem, leaf and flower from GH1 and JH3 (100G data per sample). mRNA data for the remaining genomes were downloaded from the National Genomics Data Center database. Reads were aligned to their respective genomes using Minimap2 (v2.26) or HISAT2 (v2.2.1) (Kim et al. 2019; Li 2018), followed by PASA and StringTie to generate full‐length sequences for de novo training in ANNEVO and Augustus (Bruna et al. 2020; Haas et al. 2003; Pertea et al. 2015). Protein sequences from grape, rice, maize, Arabidopsis, cabbage and cacao were used for homology‐based prediction using miniprot (Li 2023). Gene models were integrated using EvidenceModeler (Haas et al. 2008), and predicted proteins were functionally annotated using Swiss‐Prot and InterPro databases. To identify structural genes in the anthocyanin pathway, we annotated the genome using the gfanno pipeline (v.1.4) (Du et al. 2024). This tool employs a combined HMMSearch and BLASTP pipeline, which effectively filters out incomplete and aberrant sequences.

### 6.5 Whole‐Genome Resequencing and Variant Calling

Genomic DNA was extracted from leaf tissues of 94 samples. DNA libraries were prepared according to the manufacturer's instructions and sequenced on the DNBSEQ‐T7 platform (Berry Genomics), generating an average of 18× coverage per sample. Both new and previously published resequencing data were quality‐filtered using Trimmomatic (v0.39) with default settings (Bolger et al. 2014). Cleaned reads were aligned to the GH1 reference genome using BWA‐mem2 (v2.2.1), and duplicate reads were marked. Variant calling followed established protocols using GATK pipelines (McKenna et al. 2010): HaplotypeCaller was used to generate GVCF files for each sample, CombineGVCFs was used to merge individual GVCFs from 237 samples, and GenotypeGVCFs was used to call genotypes. Final variant filtering was performed using PLINK with the parameters ‘‐biallelic‐only ‐geno 0.01 ‐maf 0.05’.

### 6.6 Phylogenetic and Population Structure Analysis

For the population evolution analysis of the *Camellia* genus, SNPs with no missing data from 237 samples were used to construct a phylogenetic tree. Two methods were employed: the maximum likelihood‐based IQ‐TREE (v2.3.6) and the recently developed VCF2Dis, which rapidly builds distance‐based phylogenetic trees using default parameters (Minh et al. 2020; Xu et al. 2025). The resulting tree was visualised using the online tool iTOL Letunic and Bork (2021). For population structure analysis, 1 361 364 SNPs were retained after linkage disequilibrium‐based filtering and analysed using ADMIXTURE across different *K* values (Alexander et al. 2009). For the phylogenetic tree of 12 genomes, single‐copy orthologs were identified using OrthoFinder2 (v2.5.5) with default parameters (Emms and Kelly 2019). Gene sequences of each OGs were aligned using MAFFT v7.525 with the L‐INS‐i option (Katoh and Standley 2013), and poorly aligned regions were removed using TRIMAL v1.5.0 with the ‘‐automated 1’ setting (Capella‐Gutiérrez et al. 2009). Gene trees were constructed using IQ‐TREE (v2.3.6) with the parameter ‘‐B 5000’ for ultrafast bootstrap analysis (Minh et al. 2020), and ASTRAL v1.54 was used to infer the final species tree by combining these gene trees (Zhang et al. 2020).

### 6.7 Ancestral State Reconstruction

Ancestral states for floral colour were reconstructed using Mesquite software (v.4.01) under both maximum parsimony and maximum likelihood methods. The trait was modelled as discrete with the following: 0 for white, 1 for yellow and 2 for red/pink.

### 6.8 Gene Family Analysis

Homologous gene families across the 11 *Camellia* genomes were identified using OrthoFinder (v2.5.5). These were categorised as core (present in all 11 genomes), soft‐core (9–10 genomes), dispensable (2–8 genomes) and unique (present in only one genome). Genes without homologues and tandem duplications were defined as orphan genes.

### 6.9 SV Identification and Validation

Whole‐genome alignments between the 10 genomes and the GH1 reference genome were performed using nucmer (v4.0.0rc1) with parameters ‐c 100 ‐l 40 (Marçais et al. 2018). Alignments were filtered using delta‐filter (−m ‐i 90 ‐l 100), and coordinate files were generated using show‐coords (‐THrd). Divergent sequences within species‐specific regions were initially called using show‐diff. Following this, the putative variants were aligned to the reference genome via minimap2, and only those alignments with < 80% sequence coverage were retained for downstream analysis. SVs were identified using both SyRI (v1.7.0) and SVIM‐asm with default settings (Goel et al. 2019); Heller and Vingron (2021) and only SVs supported by both tools were retained. In addition, SVs and TEs were considered overlapping if > 50% of the SV sequence coincided with a TE. For SV validation, Integrative Genomics Viewer (v2.16.0) was used to examine a subset of randomly selected SVs. TGS reads were mapped to the genomes, and the SV regions were manually inspected.

### 6.10 Graph Genome Construction and Population SVs Calling

A graph‐based pangenome was constructed using the vg graph tool (v1.56.0), incorporating merged insertion (INS) and deletion (DEL) structural variants into the linear GH1 reference genome (Garrison et al. 2018). XG and GCSA index files were generated using the vg index tool with default settings. Next‐generation sequencing (NGS) data from 176 samples were mapped to the graph genome using vg map. Low‐quality alignments were filtered with vg pack using parameters ‐Q 5 ‐s 5. Structural variants were genotyped for each sample using vg call with parameters ‐a ‐s. SV calls from all samples were then merged into a single VCF file using bcftools (v1.13) (Danecek et al. 2021).

### 6.11 Anthocyanin Measurement

Anthocyanin identification and quantification followed the method described in our previous study (Fan et al. 2023). Briefly, fresh petal powder was incubated in 5 mL of extraction buffer (methanol–water–formic acid–trifluoroacetic acid, 70:27:2:1, *v/v*) for 24 h in the dark. The extract was filtered through a 0.22‐μm membrane and stored at −20°C. The chromatographic elution gradient was: 0 min at 22% B, 15 min at 28% B and 35 min at 68% B, with detection at 525 nm. A cyanidin‐3‐O‐*β*‐glucoside (Cy3G) standard (Sigma, St. Louis, MO, USA) was used for quantification.

### 6.12 Transcriptome Analysis

Total RNA was extracted from petal samples stored in liquid nitrogen using the RN38 Kit (Aidlab, Beijing), following the manufacturer's protocol. NGS libraries were prepared and sequenced on an Illumina platform (Berry Genomics, Beijing). Low‐quality reads and adapters were trimmed with Trimmomatic (v0.39) using default settings. Filtered reads were mapped to reference genomes using HISAT2 with default parameters. Gene expression levels were quantified using featureCounts (Liao et al. 2014), and differential gene expression analysis was performed using the *limma* package (Ritchie et al. 2015).

### 6.13 *Agrobacterium*‐Mediated Transient Transformation

The full‐length *MYB60* CDS was cloned into the pCAMBIA1300 vector to generate the *CcMYB60*‐GFP expression construct, which was transformed into *Agrobacterium tumefaciens* strain GV3101. After centrifugation, the bacterial pellet was resuspended in infiltration buffer to an OD_600_ of 1.0. Petal discs were vacuum‐infiltrated at −80 kPa for 3 min, repeated twice. Following infiltration, the petals were incubated in the dark at 18°C for 2 days, then transferred to light conditions for an additional 2 days before phenotypic analysis. Each treatment included three independent biological replicates, with ~20 petal discs per replicate.

### 6.14 Luciferase Report Experiment

The target sequences were amplified by PCR and inserted into the LUC luciferase reporter vector using a ClonExpress ultra one step cloning kit (Vazyme, Nanjing). The resulting constructs were introduced into *A. tumefaciens* GV3101 (pSoup) and transiently expressed in *Nicotiana benthamiana* leaves via Agrobacterium‐mediated infiltration. Firefly and Renilla luciferase activities were measured using the Bio‐Lite Luciferase Assay System (Vazyme, Nanjing).

## Author Contributions

M.F. conceived and designed the study, M.F. performed data analysis and wrote the manuscript. Y.Q., H. Jiang and Y.Z. performed the experiments. X.L. prepared the samples. Y.W. and X.L. revised the paper. All authors read and approved the final paper.

## Conflicts of Interest

The authors declare no conflicts of interest.

## Supporting information


**Figure S1:** Phylogenetic tree of 237 Camellia accession was inferred by iqtree based on SNP data. *Tucheria hexalocularia* and *Polyspora speciosa* were identified as outgroups.
**Figure S2:** Phylogenetic tree of 237 Camellia accession was inferred by VCF2DIS based on SNP data. *Tucheria hexalocularia* and *Polyspora speciosa* were identified as outgroups.
**Figure S3:** Principal component analysis of *Camellia* accessions. PC1 and PC2 account for 22.14% and 15.55% of the total variation respectively.
**Figure S4:** Ancestral state reconstruction for flower colour of *Camellia*, performed using both the maximum parsimony method (above the node of the tree) and the maximum likelihood method (below the node of the tree) for the backbone of nuclear phylogeny. The pie diagrams in the internal nodes represent the most likely ancestral character states and the relative probabilities of each alternative state. The red arrows point to the common ancestor nodes of *Camellia*, showing the inferred ancestral character states of flower colour.
**Figure S5:** Heatmap showing different anthocyanin relative content in red‐flowered species. Data were normalised to the mean of each row to highlight variations in abundance profiles. Red and blue indicate high and low frequency respectively.
**Figure S6:** (a, b) Phenotypic characteristics of two newly sequenced species: *C. hongkongensis* (a) and *C. chrysanthoides* (b). (c, d) Genome size estimation of *C. hongkongensis* (GH1) and *C. chrysanthoides* (JH3) via flow cytometry, using *Solanum lycopersicum* (Heinz1706; 0.88 Gb) as an internal reference standard.
**Figure S7:** Assessment of genome assemblies. (a) (b) The distribution of 21‐bp Kmer of the corresponding genome. The Kmer abundance is used to calculate the estimated genome size. (c) (d) Each plot displays the copy number spectrum of an individual genome with its corresponding quality value. (e) (f) The heat map shows the intensity signals of Hi‐C chromosome interaction.
**Figure S8:** (a) The phylogenetic relationship and estimation of divergence times of *Camellia* and *A. chinensis* , *R. simsii*, *V. vinifera* , *T. cacao* , *A. trichopoda* . (b) The number of pairwise shared and still‐intact fl‐LTRs across species. The reading direction is column to row.
**Figure S9:** Representative synteny between GH1 and the other camellia assemblies. Gene synteny was assessed using the MCScanX program to identify collinear blocks of syntenic gene pairs.
**Figure S10:** Proportion of transposable elements within the identified structural variants. Bar graph showing the mean proportion (± SEM) of each transposable element type (Copia, Gypsy, TIR, helitron and other) aggregated from all analysed structural variants (SVs) (*n* = 10 samples). Different letters indicate significant differences at *p* < 0.05.
Figure. S11 Examples of structural variant (SV) validation using Integrative Genomics Viewer (IGV) were performed manually. The randomly selected deletion in the JH3 genome, with the long‐reads of JH3 being mapped to the GH1 genome.
**Figure S11:** Examples of structural variant (SV) validation using Integrative Genomics Viewer (IGV) were performed manually. The randomly selected deletion in the JH3 genome, with the long‐reads of JH3 being mapped to the GH1 genome.
**Figure S12:** Phylogenetic tree of 176 *Camellia* accession based on structural variant (SV) data obtained from a graph‐based genome. Different colour indicates the distribution of the five sections.
**Figure S13:** Venn diagram showing the number of differentially expressed genes (DEGs) across red, white and yellow petal.

**Data S1:** Supporting Information

## Data Availability

The data that support the findings of this study are available on request from the corresponding author. The data are not publicly available due to privacy or ethical restrictions.
