# Supplementary material for: Transposable Element‐Mediated Structural Variation Drives Flower Colour Diversification in Camellia
Source: Plant Biotechnol J. 2025 Nov 6;24(3):1725–39. doi: 10.1111/pbi.70442 (PMC12946509; doi:10.1111/pbi.70442)
Supplement: Supplementary file 1 — Figure S1: Phylogenetic tree of 237 Camellia accession was inferred by iqtree based on SNP data. Tucheria hexalocularia and Polyspora speciosa were identified as outgroups. Figure S2: Phylogenetic tree of 237 Camellia accession was inferred by VCF2DIS based on SNP data. Tucheria hexalocularia and Polyspora speciosa were identified as outgroups. Figure S3: Principal component analysis of Camellia accessions. PC1 and PC2 account for 22.14% and 15.55% of the total variation respectively. Figure S4: Ancestral state reconstruction for flower colour of Camellia, performed using both the maximum parsimony method (above the node of the tree) and the maximum likelihood method (below the node of the tree) for the backbone of nuclear phylogeny. The pie diagrams in the internal nodes represent the most likely ancestral character states and the relative probabilities of each alternative state. The red arrows point to the common ancestor nodes of Camellia, showing the inferred ancestral character states of flower colour. Figure S5: Heatmap showing different anthocyanin relative content in red‐flowered species. Data were normalised to the mean of each row to highlight variations in abundance profiles. Red and blue indicate high and low frequency respectively. Figure S6: (a, b) Phenotypic characteristics of two newly sequenced species: C. hongkongensis (a) and C. chrysanthoides (b). (c, d) Genome size estimation of C. hongkongensis (GH1) and C. chrysanthoides (JH3) via flow cytometry, using Solanum lycopersicum (Heinz1706; 0.88 Gb) as an internal reference standard. Figure S7: Assessment of genome assemblies. (a) (b) The distribution of 21‐bp Kmer of the corresponding genome. The Kmer abundance is used to calculate the estimated genome size. (c) (d) Each plot displays the copy number spectrum of an individual genome with its corresponding quality value. (e) (f) The heat map shows the intensity signals of Hi‐C chromosome interaction. Figure S8: (a) The phylogenetic relationship and [file PBI-24-1725-s001.docx]

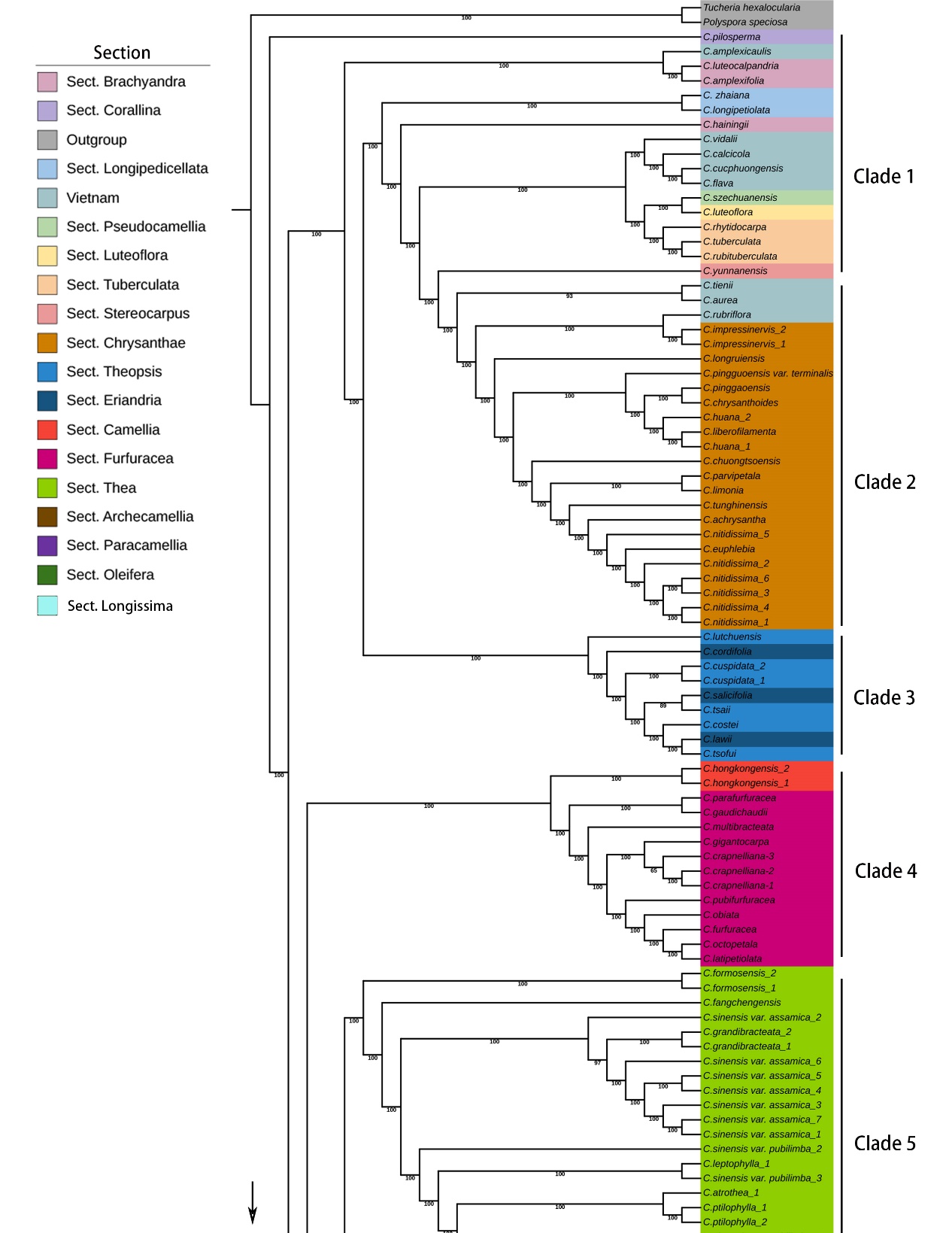


Fig.S1 Phylogenetic tree of 237 Camellia accession was inferred by iqtree based on SNP data. *Tucheria hexalocularia* and *Polyspora speciosa* were identified as outgroups.


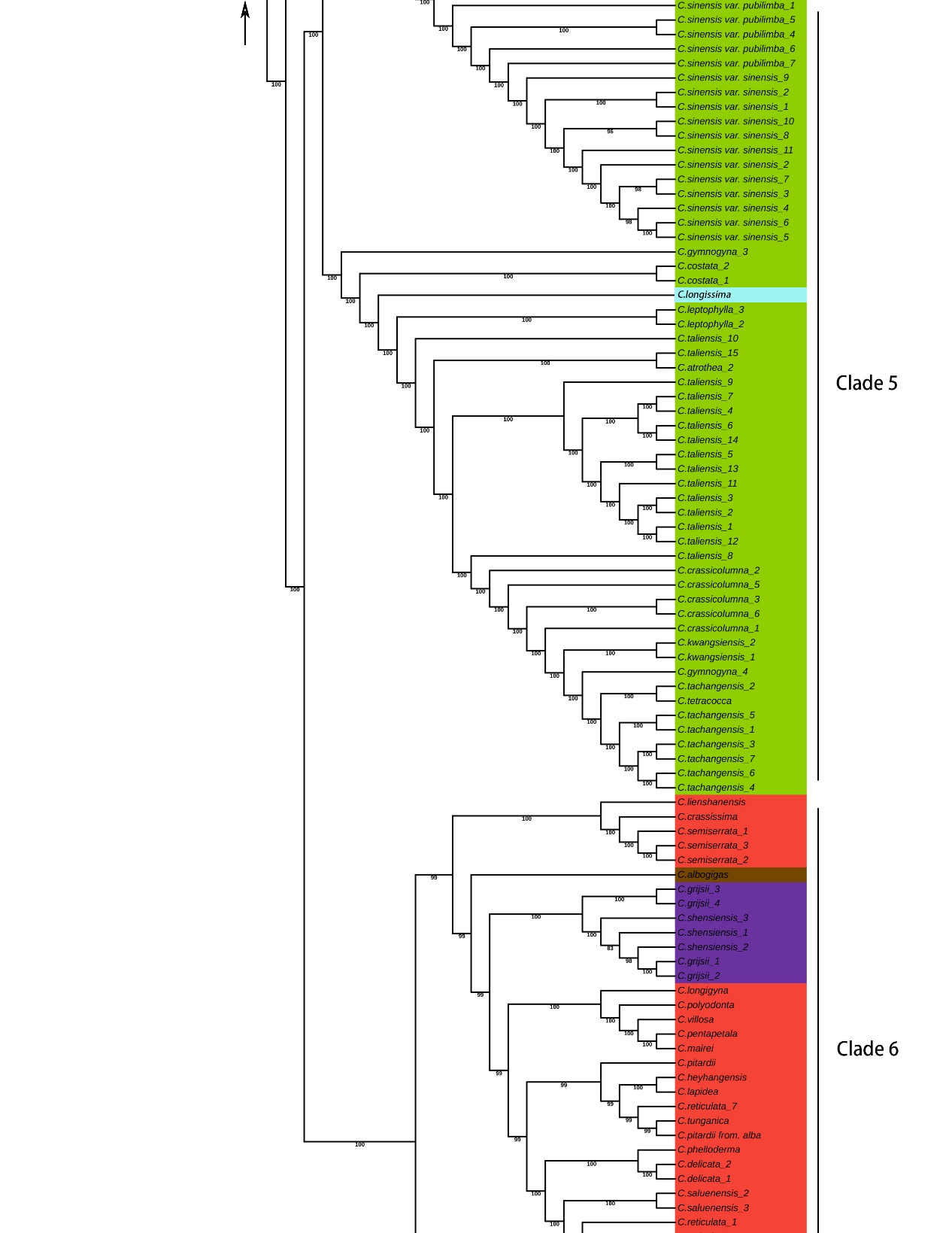


Fig.S1 Phylogenetic tree of 237 Camellia accession was inferred by iqtree based on SNP data. *Tucheria hexalocularia* and *Polyspora speciosa* were identified as outgroups.


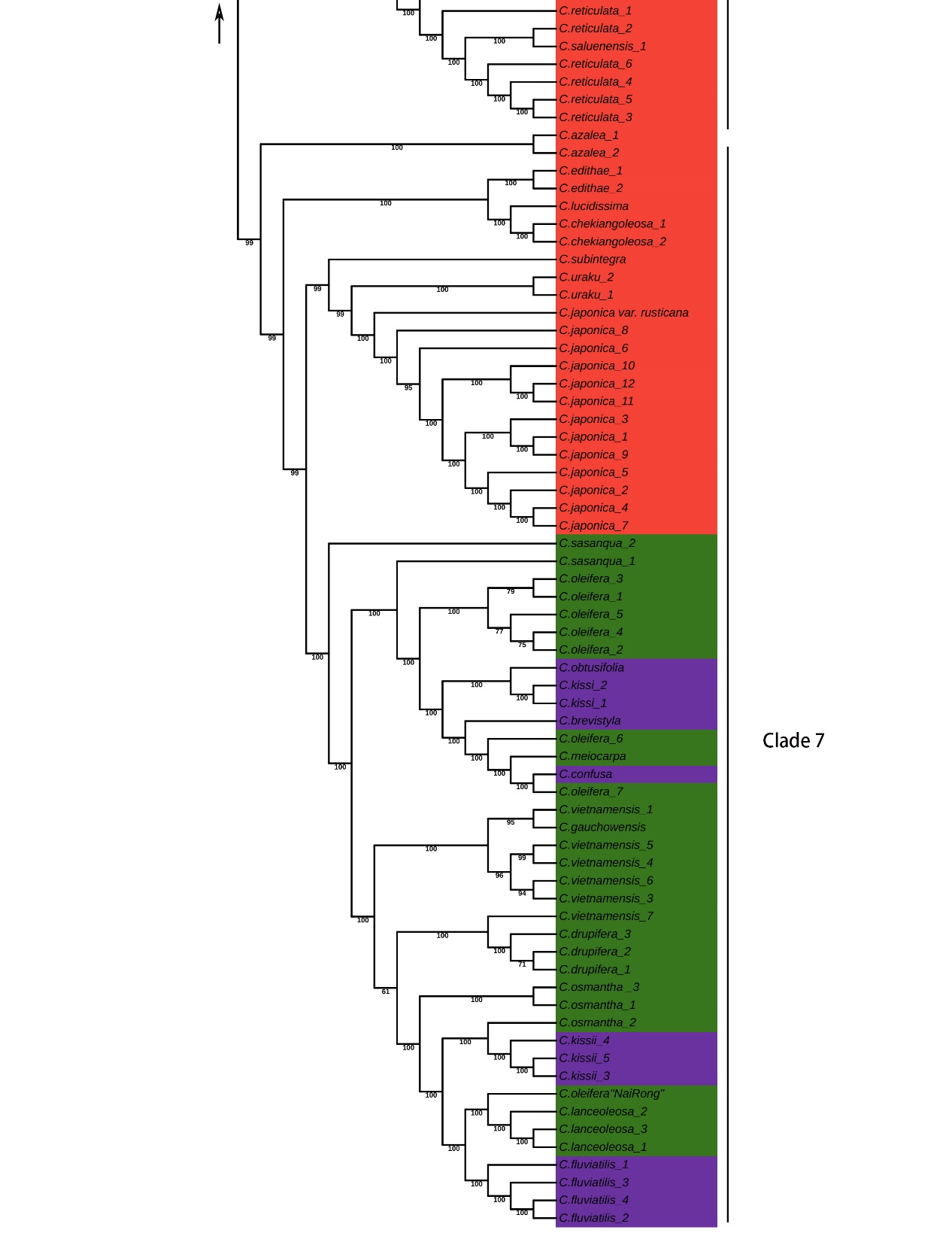


Fig.S1 Phylogenetic tree of 237 Camellia accession was inferred by iqtree based on SNP data. *Tucheria hexalocularia* and *Polyspora speciosa* were identified as outgroups.


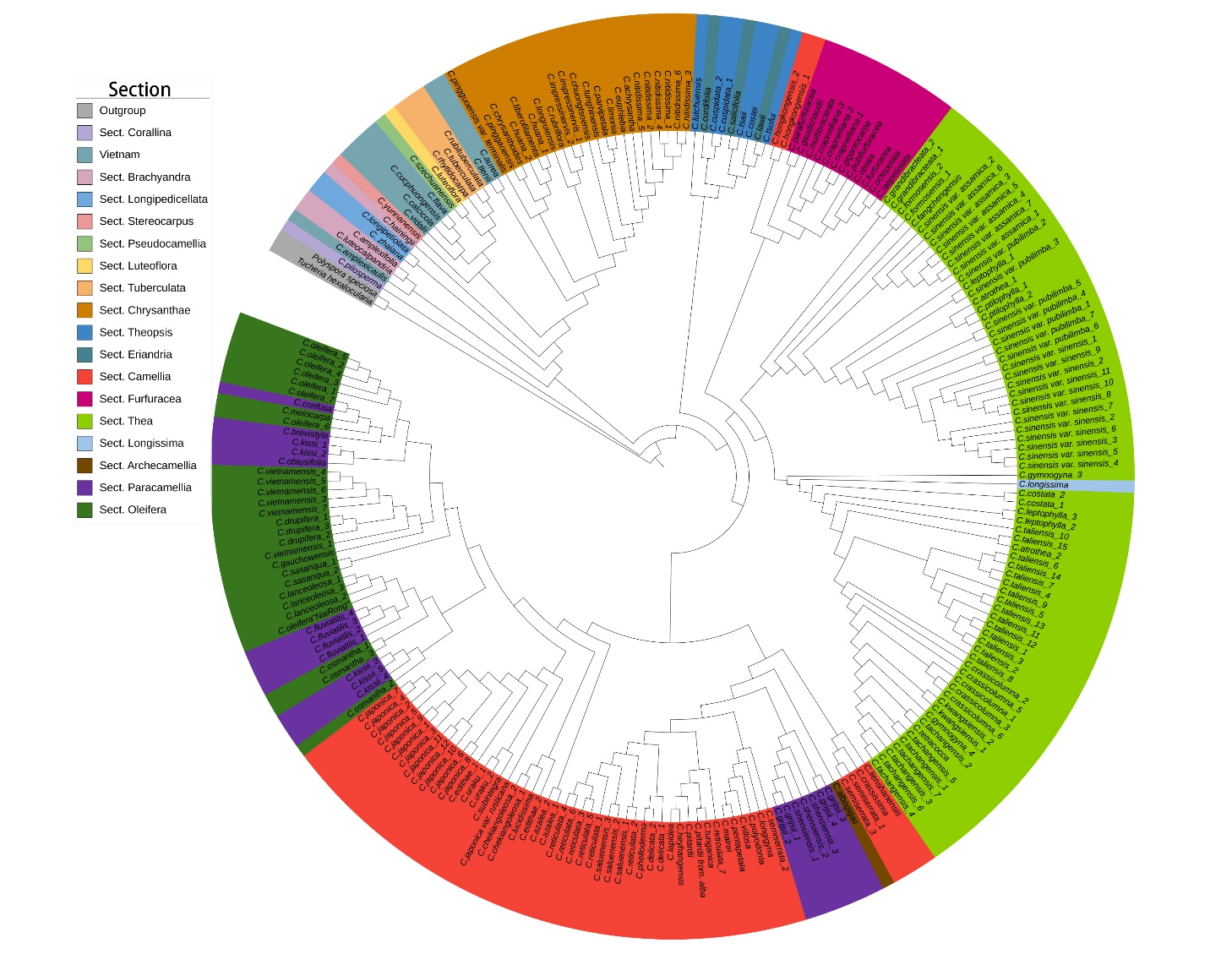


Fig.S2 Phylogenetic tree of 237 Camellia accession was inferred by VCF2DIS based on SNP data. *Tucheria hexalocularia* and *Polyspora speciosa* were identified as outgroups.


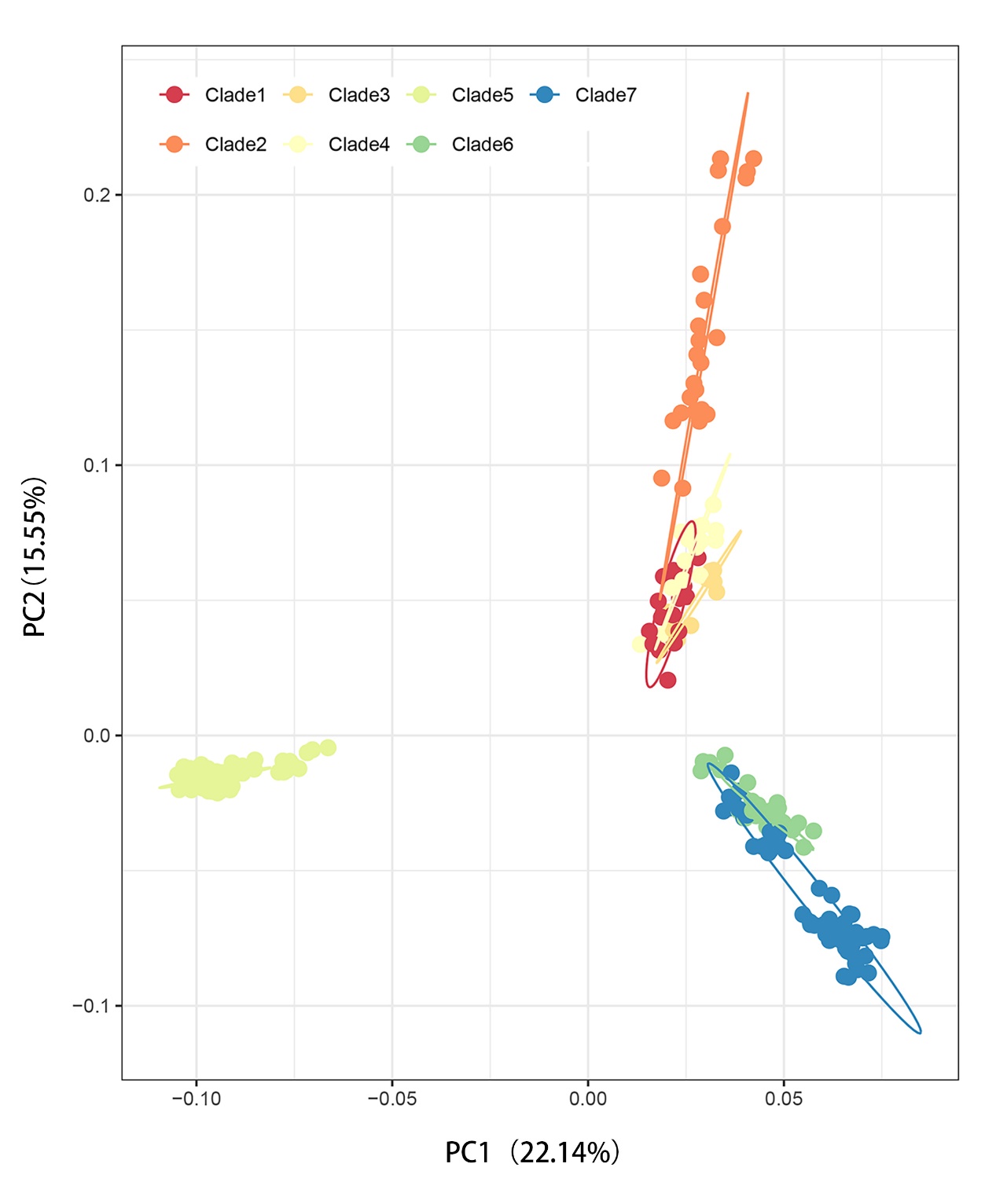


Fig.S3 Principal component analysis of *Camellia* accessions. PC1 and PC2 account for 22.14% and 15.55% of the total variation, respectively.


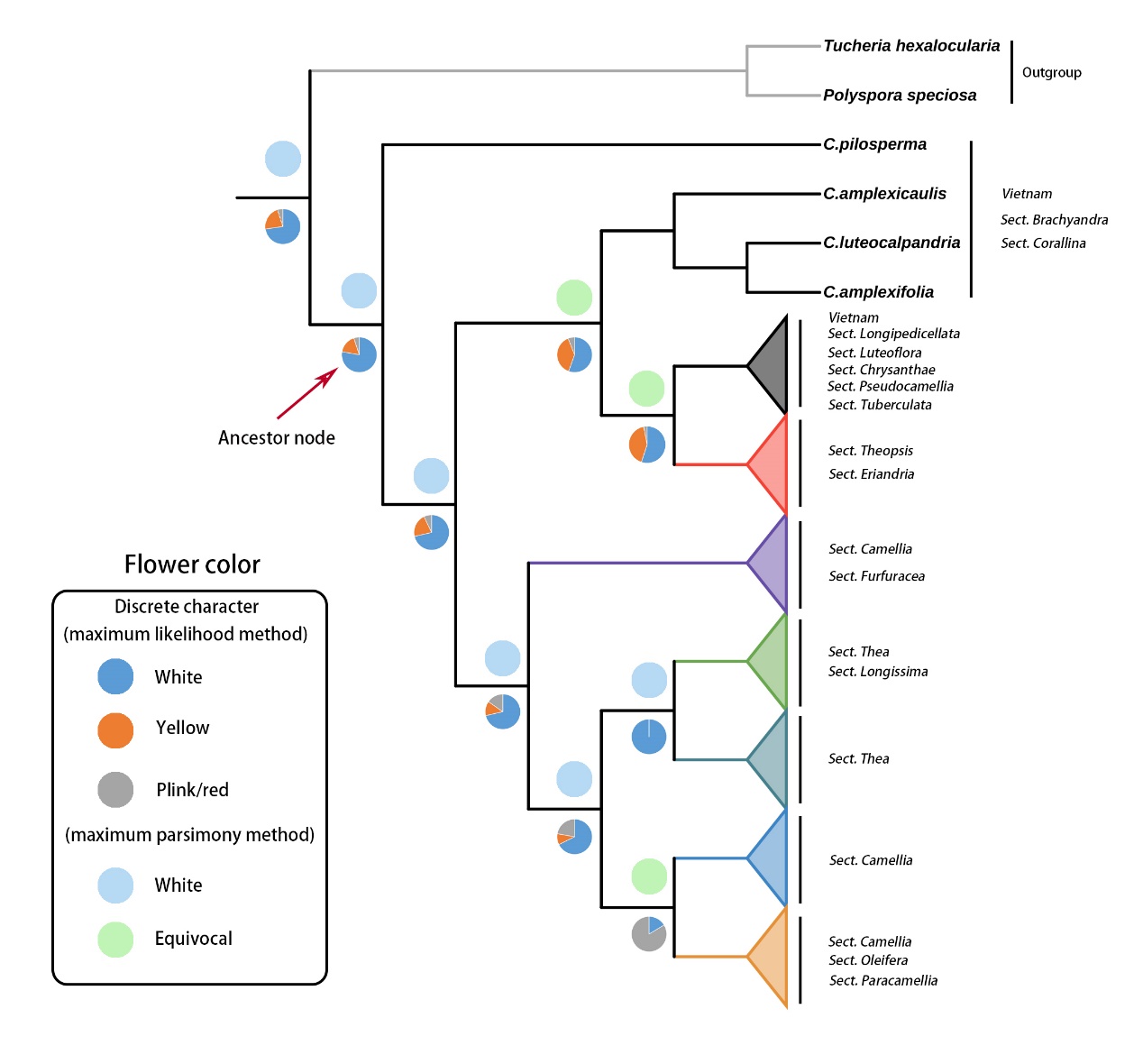


Fig.S4 Ancestral state reconstruction for flower color of *Camellia*, performed using both the maximum parsimony method (above the node of the tree) and the maximum likelihood method (below the node of the tree) for the backbone of nuclear phylogeny. The pie diagrams in the internal nodes represent the most likely ancestral character states and the relative probabilities of each alternative state. The red arrows point to the common ancestor nodes of *Camellia*, showing the inferred ancestral character states of flower color.


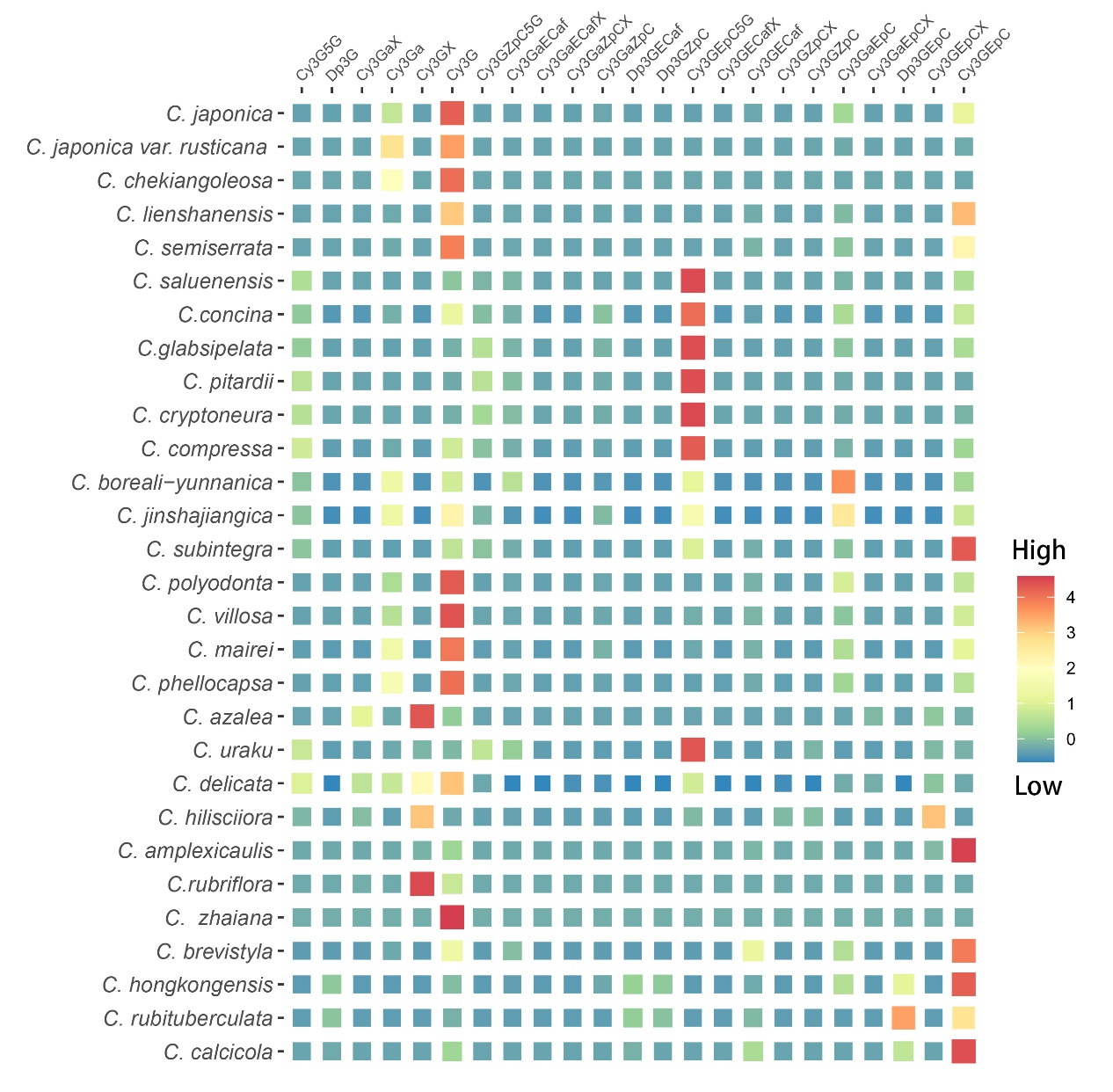


Fig.S5 Heatmap showing different anthocyanin relative content in red-flowered species. Data were normalized to the mean of each row to highlight variations in abundance profiles. Red and blue indicate high and low frequency, respectively.


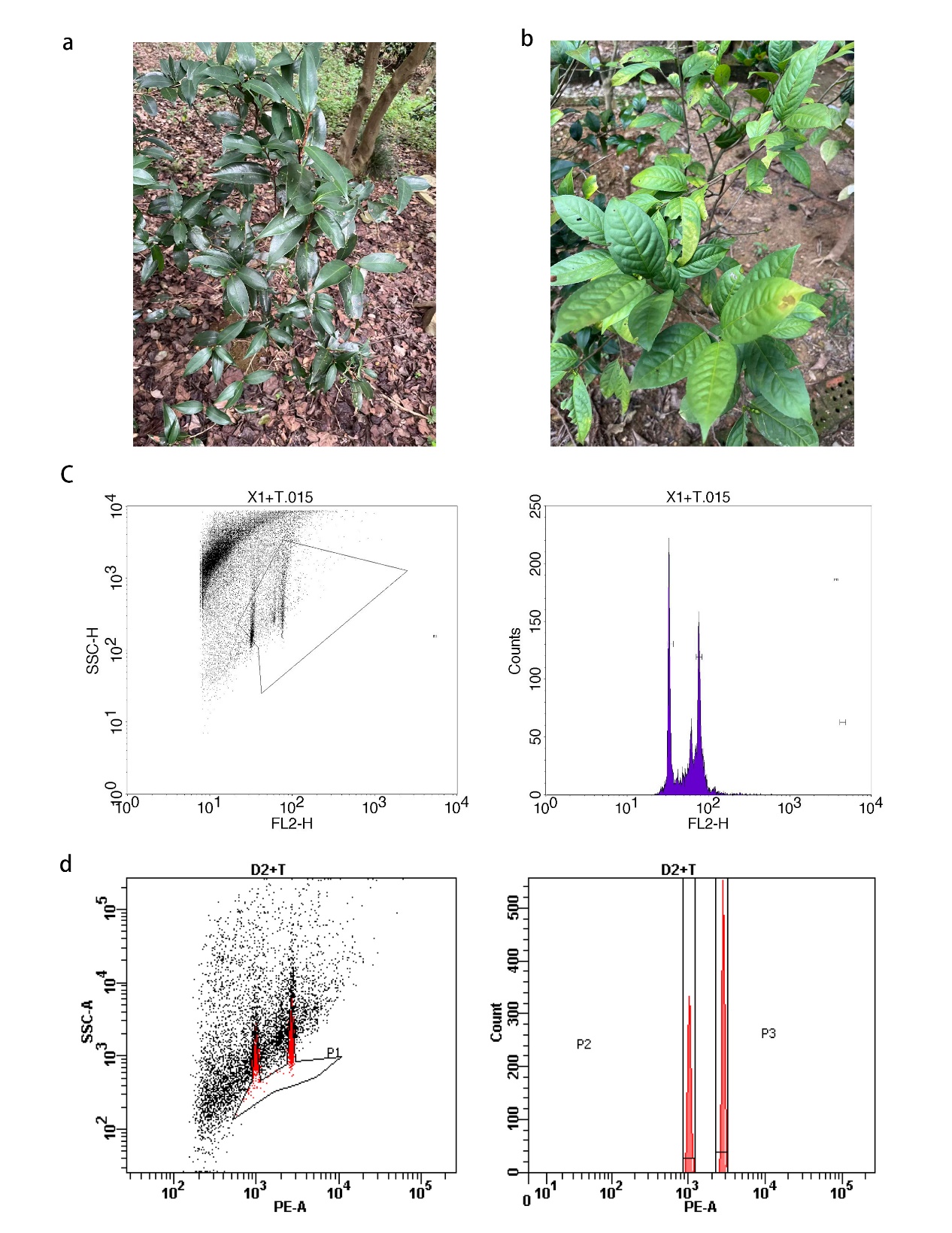


Fig.S6 (a, b) Phenotypic characteristics of two newly sequenced species: *C. hongkongensis*(a) and *C. chrysanthoides* (b). (c, d) Genome size estimation of *C. hongkongensis* (GH1) and *C. chrysanthoides* (JH3) via flow cytometry, using *Solanum lycopersicum* (Heinz1706; 0.88 Gb) as an internal reference standard.


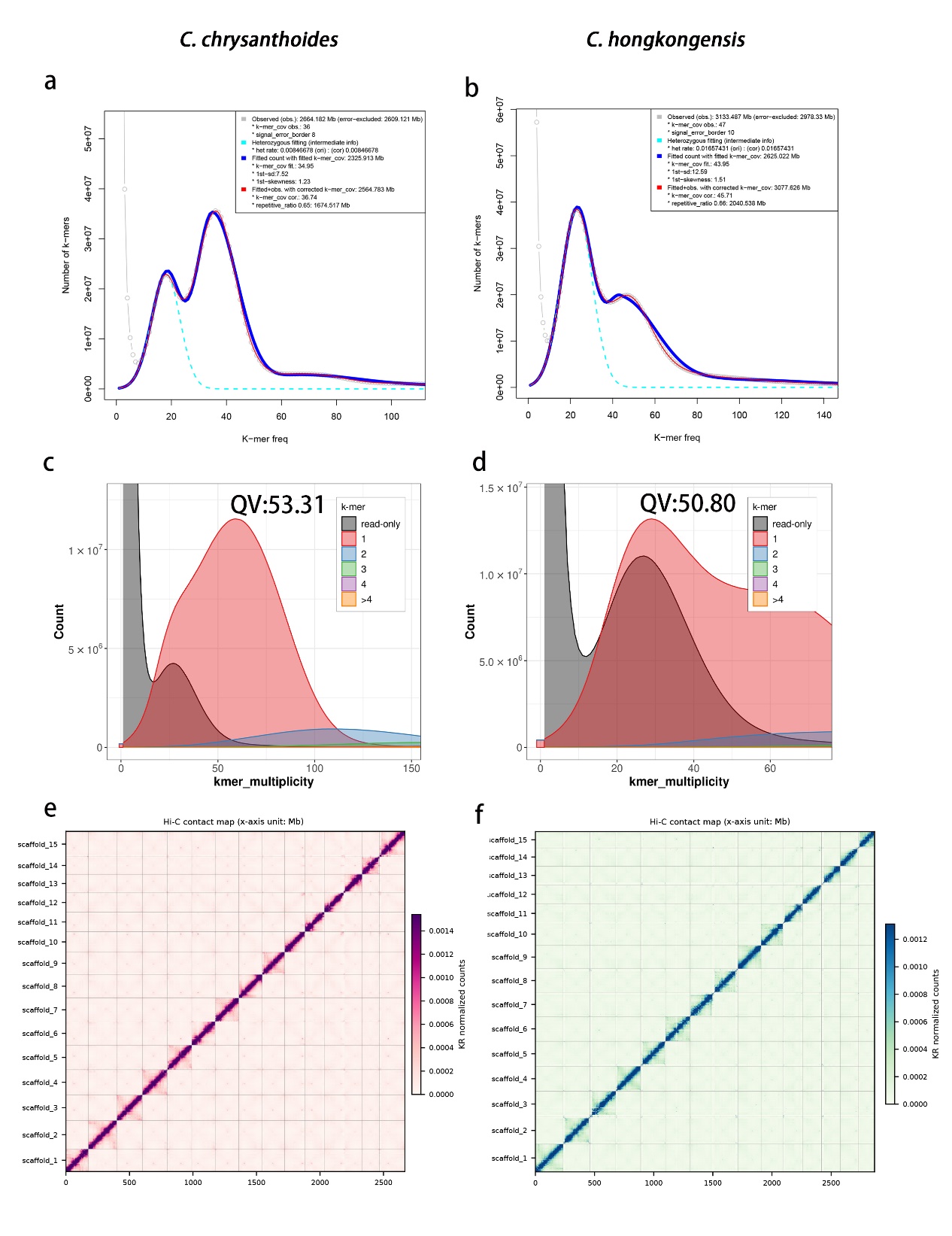


Fig.S7 Assessment of genome assemblies. (a)(b) The distribution of 21-bp Kmer of the corresponding genome. The Kmer abundance is used to calculate the estimated genome size. (c)(d) Each plot displays the copy number spectrum of an individual genome with its corresponding quality value. (e)(f) The heat map shows the intensity signals of Hi-C chromosome interaction.


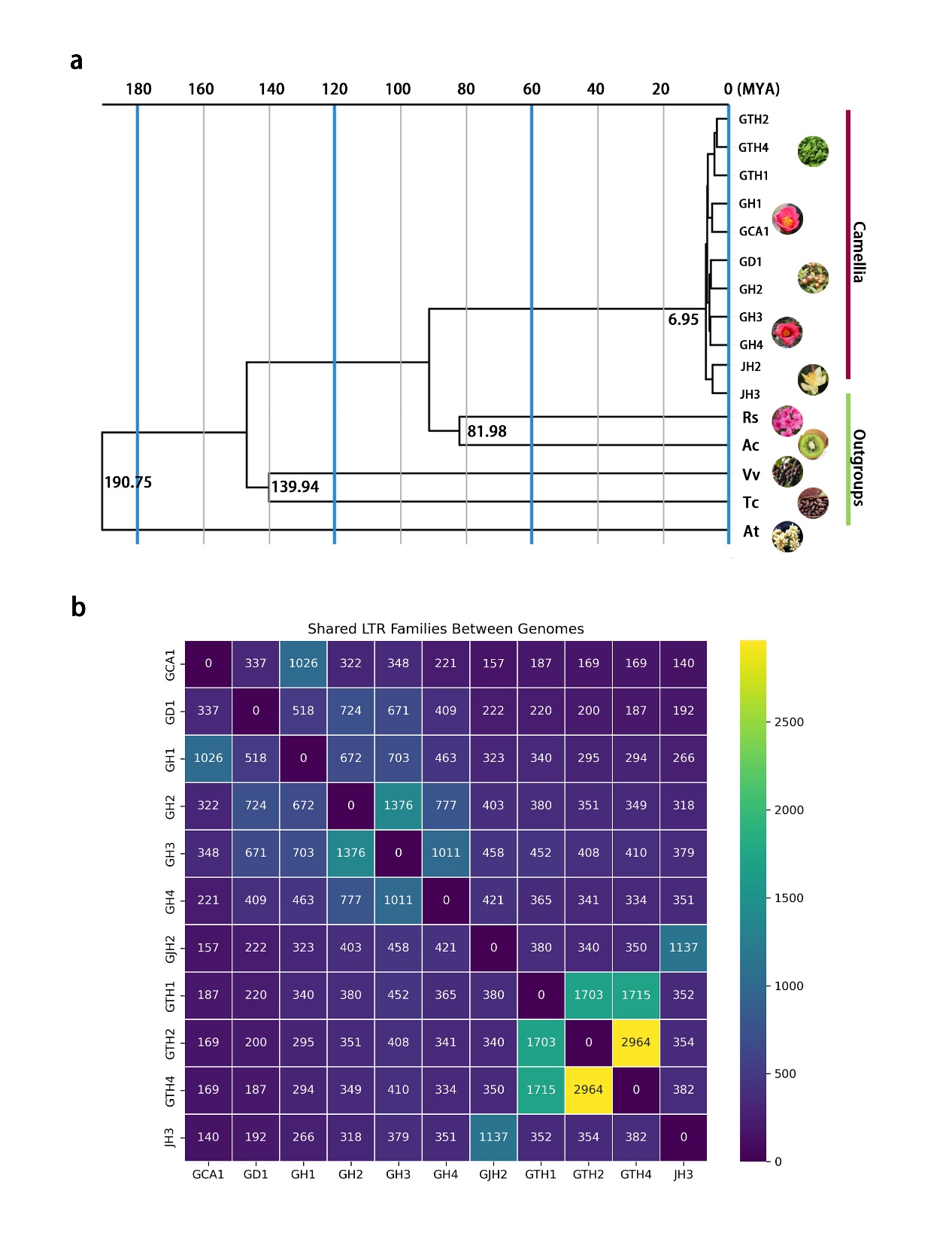


Fig.S8 (a) The phylogenetic relationship and estimation of divergence times of *Camellia* and *A. chinensis*, *R. simsii*, *V. vinifera*, *T. cacao*, *A. trichopoda*. (b) The number of pairwise shared and still-intact fl-LTRs across species. The reading direction is column to row.


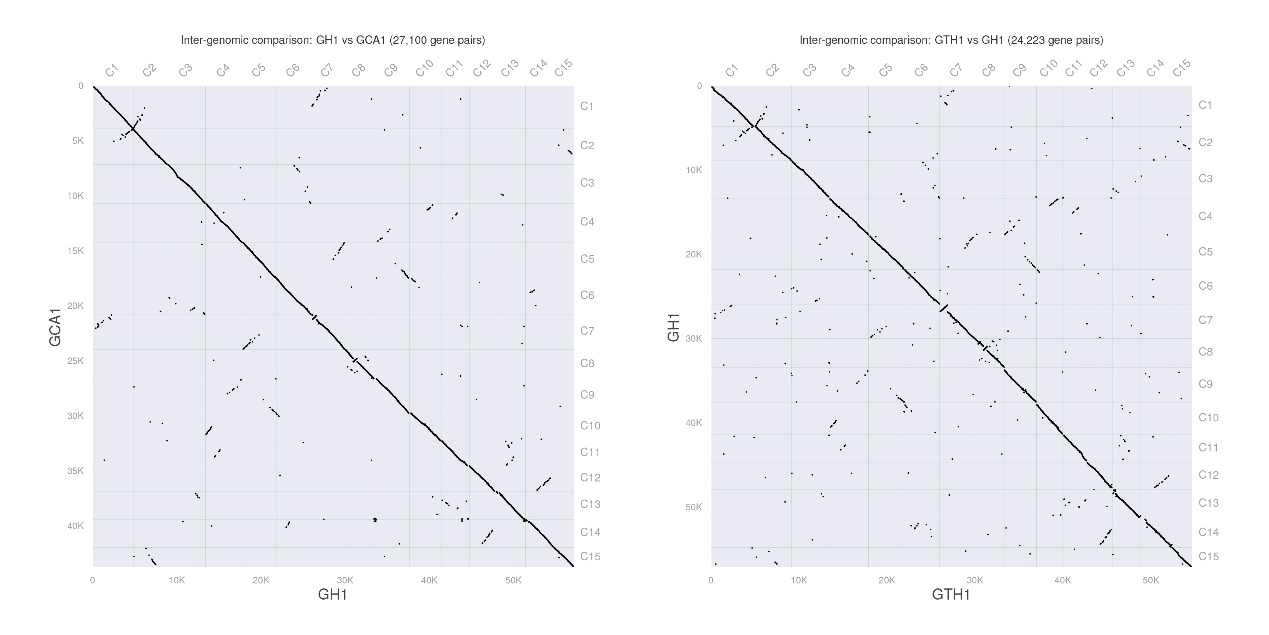


Fig.S9 Representative synteny between GH1 and the other camellia assemblies. Gene synteny was assessed using the MCScanX program to identify collinear blocks of syntenic gene pairs.


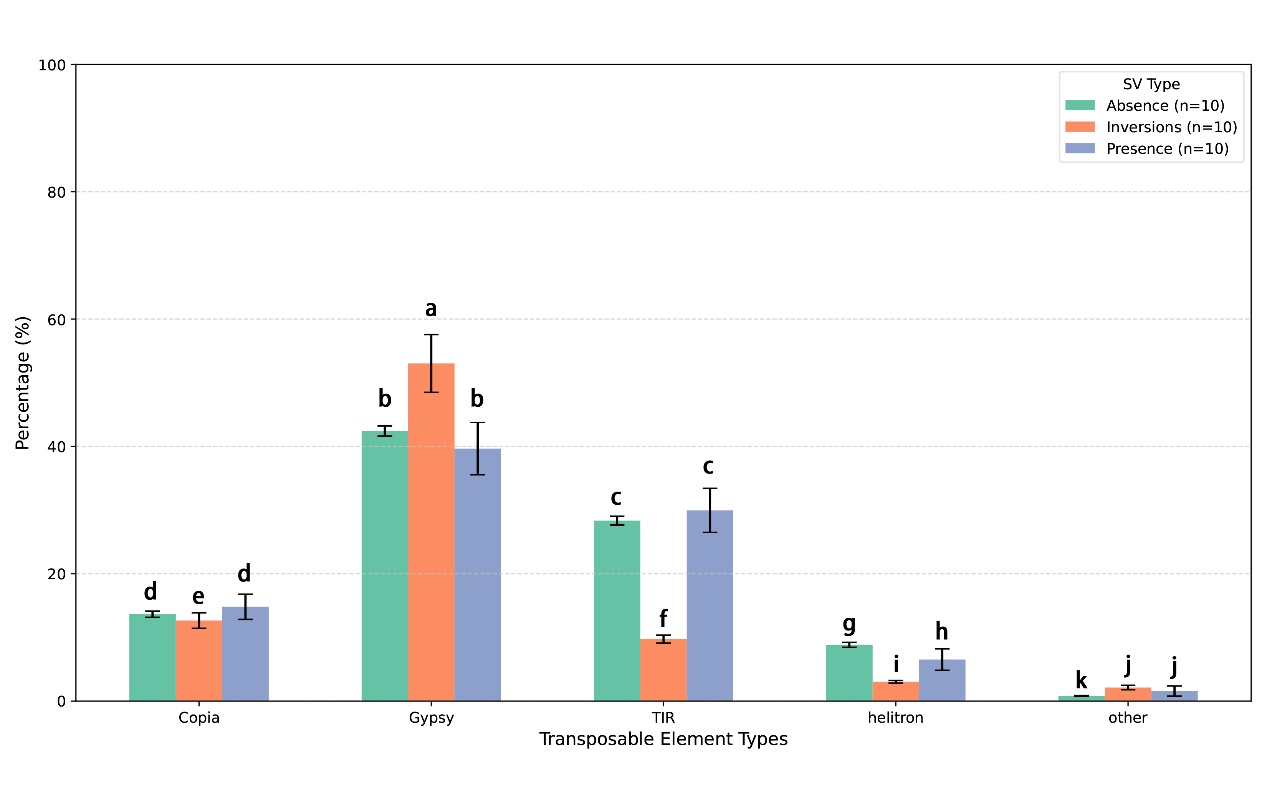


Fig.S10 Proportion of transposable elements within the identified structural variants. Bar graph showing the mean proportion (± SEM) of each transposable element type (Copia, Gypsy, TIR, helitron, and other) aggregated from all analyzed structural variants (SVs) (n = 10 samples). Different letters indicate significant differences at *p* value < 0.05.


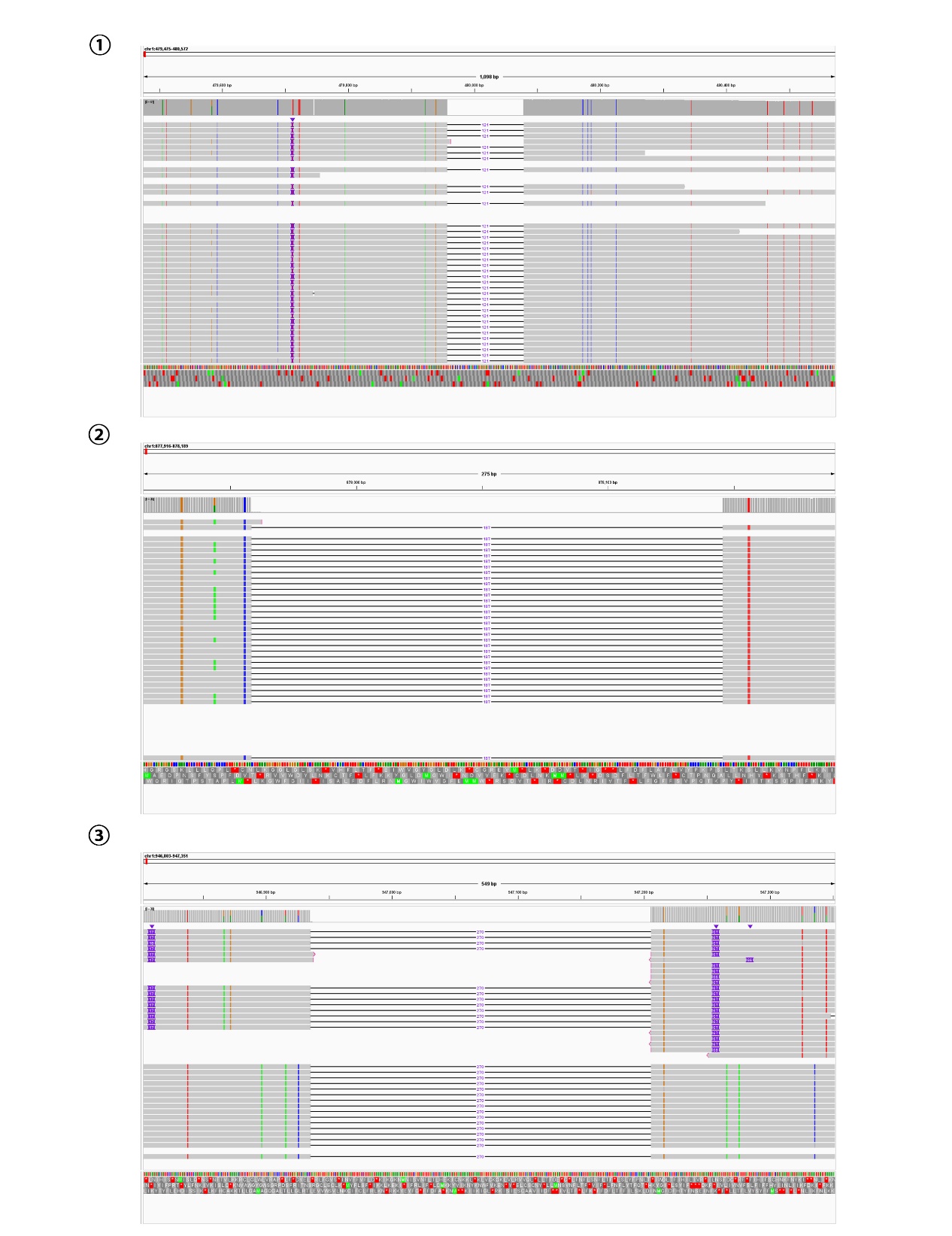


Fig.S11 Examples of structural variant (SV) validation using Integrative Genomics Viewer (IGV) were performed manually. The randomly selected deletion in the JH3 genome, with the long-reads of JH3 being mapped to the GH1 genome.


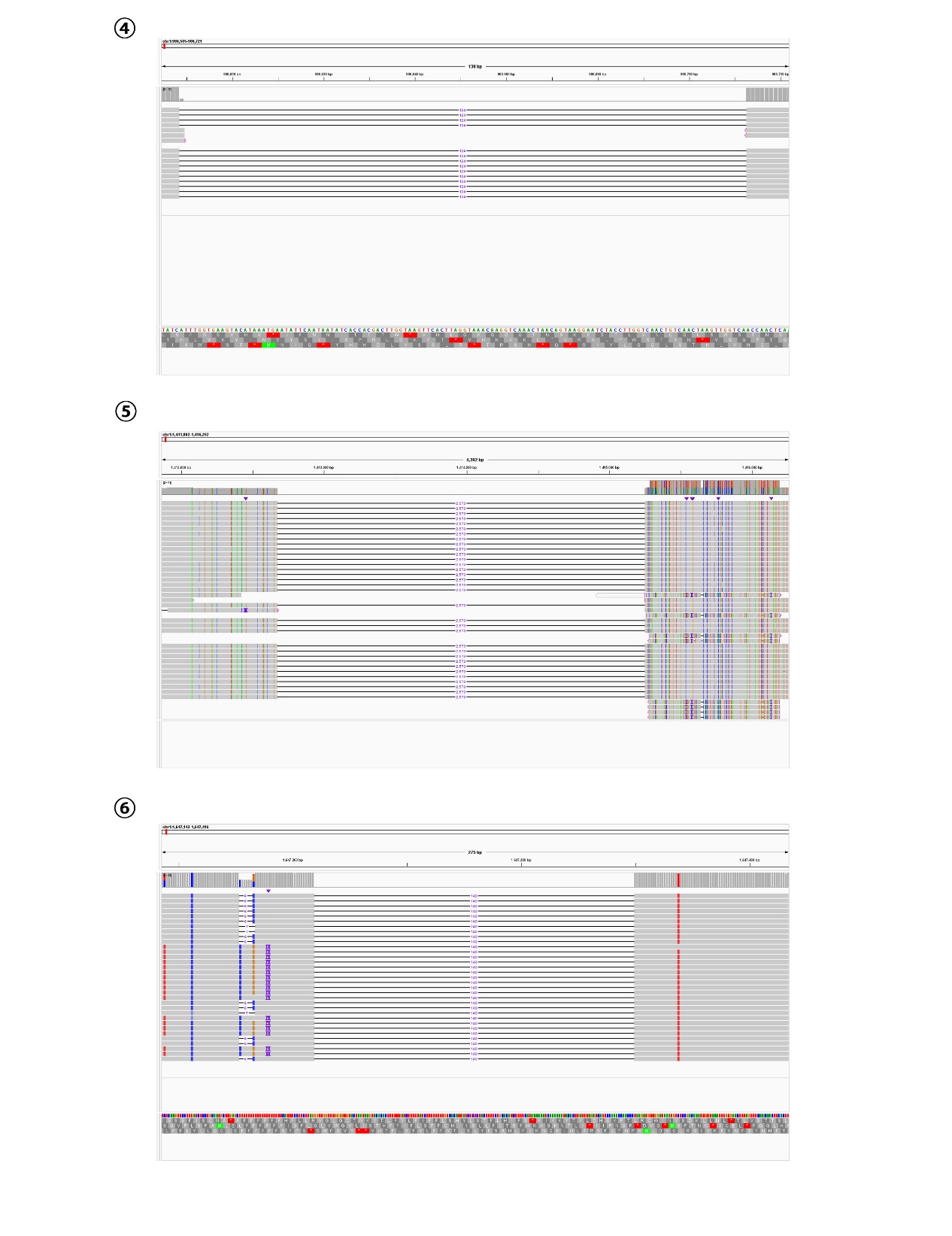


Fig.S11 Examples of structural variant (SV) validation using Integrative Genomics Viewer (IGV) were performed manually. The randomly selected deletion in the JH3 genome, with the long-reads of JH3 being mapped to the GH1 genome.


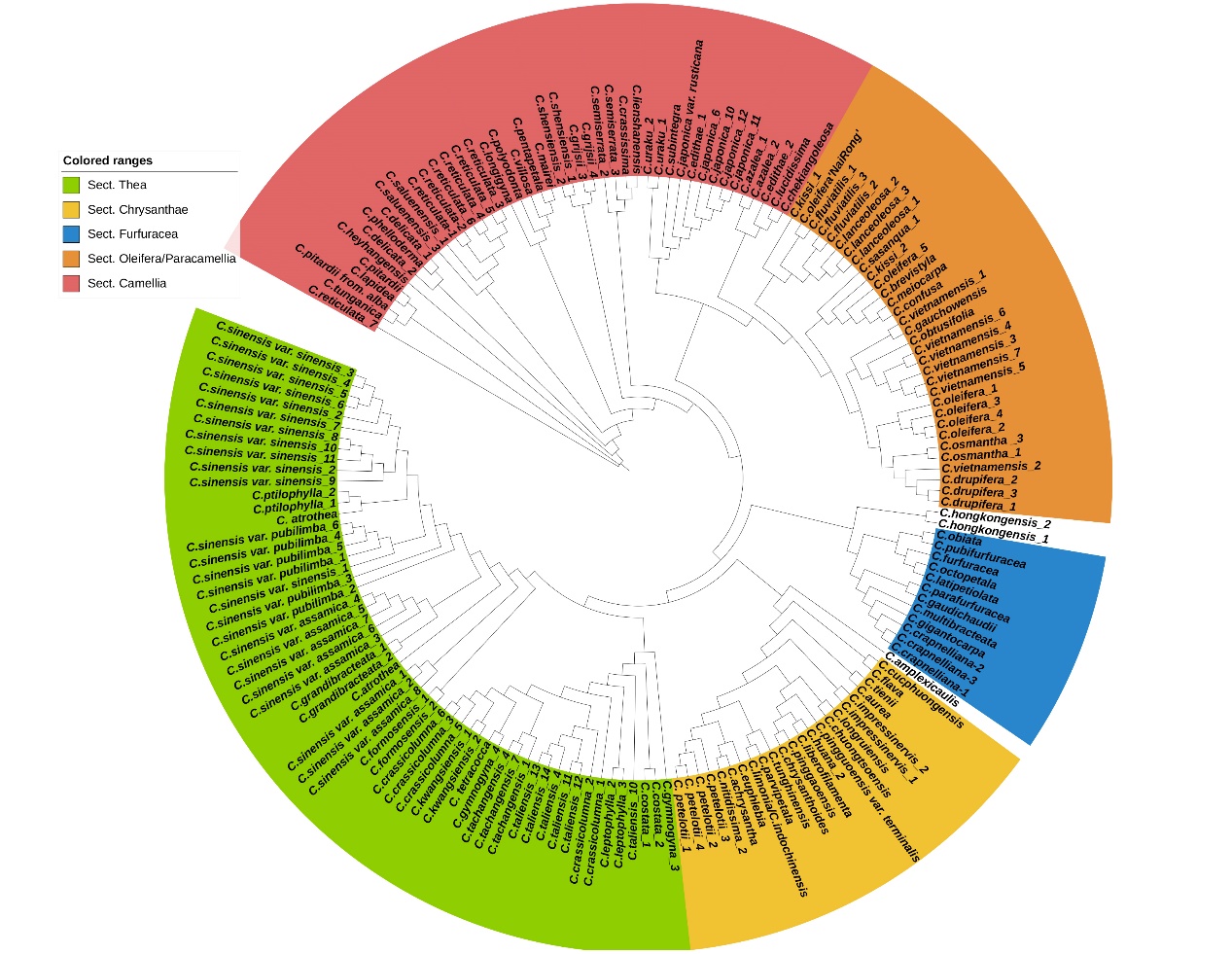


Fig.S12 Phylogenetic tree of 176 *Camellia* accession based on structural variant (SV) data obtained from a graph-based genome. Different color indicates the distribution of the five sections.


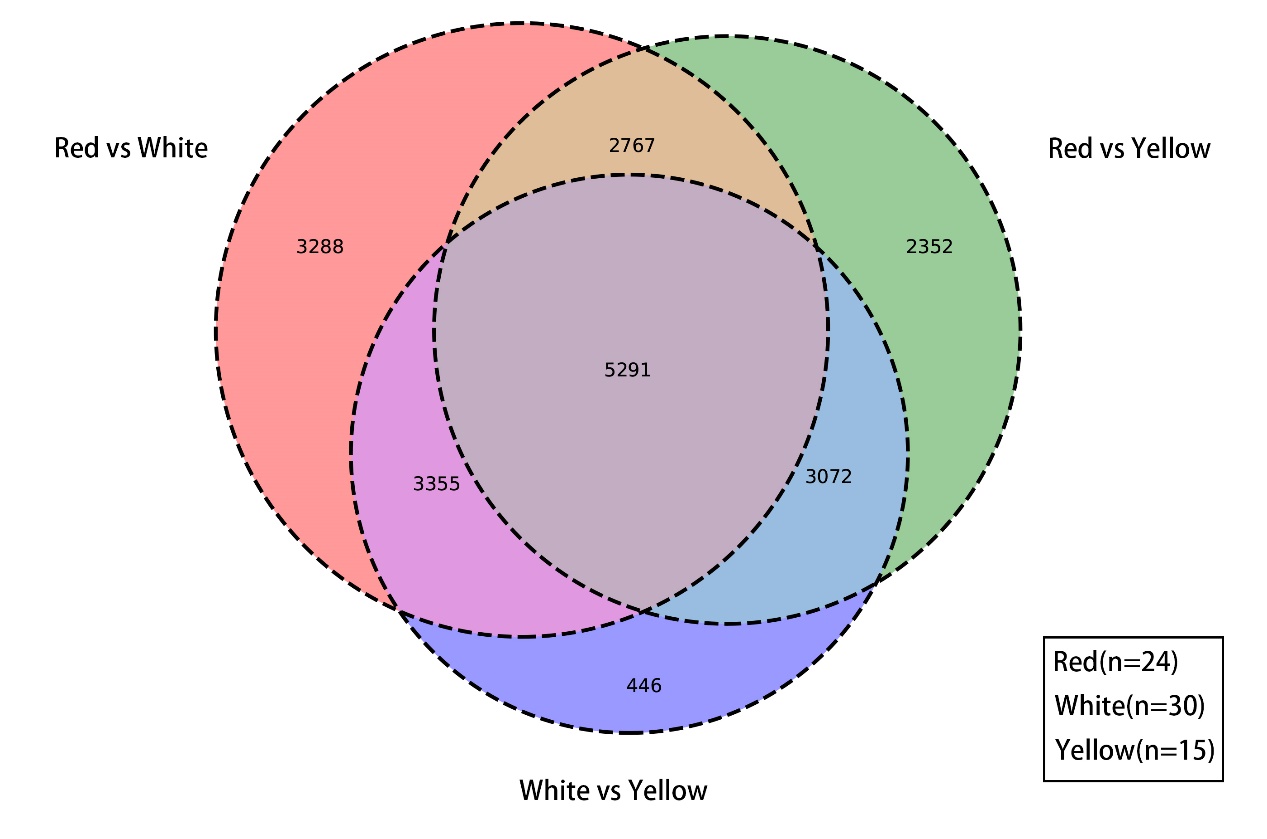


Fig.S13 Venn diagram showing the number of differentially expressed genes (DEGs) across red, white, and yellow petal.
